# Cortical and Subcortical Grey Matter Abnormalities in White Matter Hyperintensities and Subsequent Cognitive Impairment

**DOI:** 10.1007/s12264-021-00657-0

**Published:** 2021-04-07

**Authors:** Wenhao Zhu, Hao Huang, Shiqi Yang, Xiang Luo, Wenzhen Zhu, Shabei Xu, Qi Meng, Chengchao Zuo, Yong Liu, Wei Wang, Alzheimer’s Disease Neuroimaging Initiative

**Affiliations:** 1 Department of Neurology, Tongji Hospital, Tongji Medical College, Huazhong University of Science and Technology, Wuhan, 430030 China; 2 Department of Radiology, Tongji Hospital, Tongji Medical College, Huazhong University of Science and Technology, Wuhan, 430030 China; 3 Brainnetome Center and National Laboratory of Pattern Recognition, Institute of Automation, Chinese Academy of Sciences, Beijing, 100190 China; 4 School of Artificial Intelligence, Beijing University of Posts and Telecommunications, Beijing, 100876 China; 5 University of the Chinese Academy of Sciences, Beijing, 100049 China

**Keywords:** White matter hyperintensities, Cognitive impairment, Cortical thickness, Grey matter volume, Replication

## Abstract

Grey matter (GM) alterations may contribute to cognitive decline in individuals with white matter hyperintensities (WMH) but no consensus has yet emerged. Here, we investigated cortical thickness and grey matter volume in 23 WMH patients with mild cognitive impairment (WMH-MCI), 43 WMH patients without cognitive impairment, and 55 healthy controls. Both WMH groups showed GM atrophy in the bilateral thalamus, fronto-insular cortices, and several parietal-temporal regions, and the WMH-MCI group showed more extensive and severe GM atrophy. The GM atrophy in the thalamus and fronto-insular cortices was associated with cognitive decline in the WMH-MCI patients and may mediate the relationship between WMH and cognition in WMH patients. Furthermore, the main results were well replicated in an independent dataset from the Alzheimer's Disease Neuroimaging Initiative database and in other control analyses. These comprehensive results provide robust evidence of specific GM alterations underlying WMH and subsequent cognitive impairment.

**Supplementary Information:**

The online version contains supplementary material available at 10.1007/s12264-021-00657-0.

## Introduction

White matter hyperintensities (WMHs) are areas of high signals on fluid-attenuated inversion recovery (FLAIR) and T2-weighted MR images and are commonly observed in the brains of elderly individuals [1]. Converging evidence suggests that WMHs are involved in cognitive impairment in various populations, including normal aging, vascular dementia, and Alzheimer’s disease (AD) [1, 2]. Given that WMH is generally considered to represent the primary damage of cerebral small vessel disease (CSVD) due to chronic subcortical ischemia mainly involving subcortical white matter (WM) [1], previous studies concentrated on the role of WM disconnections, including aberrant WM microstructural integrity and networks in WMH progression and the cognitive decline of WMH patients [3–5]. Nevertheless, the relationship between WMH and cognitive impairment cannot be fully explained by WMH load or WM disruption, and it is still difficult to determine whether and to what extent cognitive decline exists in individual patients with WMH [1].

The grey matter (GM) is thought to play a key role in information extraction, exchange, and integration in cognitive processes. Recently, evidence has accumulated that morphological changes of GM are partly responsible for the cognitive decline in patients with WMH [6]. A few case-control studies have reported abnormalities of GM volume (GMV) or cortical thickness in subcortical vascular dementia [7–10] and subcortical vascular mild cognitive impairment (MCI) [10–13] which were mainly attributed to WMH, compared with healthy controls (HCs). Furthermore, the results of cross-sectional studies revealed that GMV (frontal or temporal cortices) mediates the relationship between WMH and cognition in non-demented individuals [14] and in a mixed sample of individuals with transient ischemic attacks, symptomatic coronary artery disease, and AD [15]. Another two cross-sectional studies showed that region-specific or global damage of cortical thickness mediates the relationship between WMH volume and cognition in CSVD [16] and older adults without dementia [17]. Overall, the results of these studies noted that specific cortical atrophy (especially GM atrophy in some frontal and temporal cortices) may be involved in the cognitive decline in WMH patients. However, the results of these studies show great divergences and there is no consensus on the characteristic structural alterations in GM underlying WMH and subsequent cognitive impairment.

The inconsistency among the few previous studies may be heterogeneity inherent to study design, different GM measurements, and various pipelines of data pre-processing and analysis. In the case-control studies that focused on GM changes in WMH patients, morphological comparisons were conducted mainly between those with cognitive impairment and HCs, and there was no information on the GM characteristics of WMH patients with normal cognition. Meanwhile, the heterogeneity of the population (i.e., a mixed sample with AD or participants with other diseases [15], different race and ethnicity, and all degrees of WMH [14, 17]) may also lead to inconsistency. These paradigms impede the exploration of a comprehensive picture of GM alterations along with the progression of WMH and subsequent cognitive impairment. In addition, it should also be noted that these studies performed GM morphometric analyses at different levels (the regional level [9, 13–17] or the voxel level [7, 8, 10–12]), using disparate methodological steps in data pre-processing and statistical analysis, which makes it difficult to directly compare the results across different studies.

In this study, we aimed to evaluate the GM structural alterations underlying cognitive decline in WMH patients. The two conventional GM measurements cortical thickness and GMV provide similar, but sometimes relatively unique, information on different types of structural changes [18–21]. And compared to the voxel-based method, GM morphometric analyses at the regional level provide direct and precise information of GM alteration in each subregion [22, 23]. Hence, we explored the role of GM alterations in WMH and subsequent cognitive impairment through studying whole-brain alteration patterns of both cortical thickness and GMV among WMH patients with MCI (WMH-MCI), WMH patients with no cognitive impairment (WMH-nCI), and healthy controls (HCs) using the fine-grained Human Brainnetome Atlas [24]. Importantly, we further conducted a series of verification analyses, including conducting GM morphometric analyses across the three groups in the ADNI database, at the voxel level, using different brain atlases, and performing group classifications, to validate the reproducibility and generalizability of the main results.

## Materials and Methods

All participants included in the in-house dataset were recruited from the outpatient service of the Department of Neurology, Tongji Hospital, between 2014 and 2016. The study was approved by the Ethics Committee of Tongji Hospital, Tongji Medical College, Huazhong University of Science and Technology (ID: TJ-C20131216). Written informed consent was given by each participant. Here we only provide a brief introduction to the participants; the details can be found in our previous study [25].

### Participants

One hundred and forty right-handed individuals, including 80 consecutive patients with moderate to severe WMH (defined as the sum of the deep WMH Fazekas score and the periventricular WMH Fazekas score ≥ 3 on FLAIR sequence images) [26, 27] and 60 HCs without moderate to severe WMH and lacunes were initially recruited. All participants underwent clinical interviews and a battery of neuropsychological tests, including the Mini-Mental State Examination (MMSE), the clinical dementia rating (CDR), the Trail Making Test (TMT), the Symbol Digit Modalities Test (SDMT), the Digit Span Test (DST), the Verbal Fluency Test (VFT), the Auditory Verbal Learning Test (AVLT), and the Hamilton depression rating scale (24-items). The neuropsychological tests were composited into three cognitive domains [28]: processing speed (TMT-A and SDMT) [29], executive function (DST backward, VFT, and TMT-B) [30], and memory (AVLT immediate recall, short delay recall, long delay recall, and long delay recognition) [31] (The details of compounded scores for each domain are shown in the Supplementary material).

As in our previous study [25], the inclusion criteria for WMH patients were: (1) moderate to severe WMH with/without lacunes; (2) no dementia based on the criteria of the Diagnostic and Statistical Manual of Mental Disorders, Fourth Edition; a CDR score ≤ 0.5; an MMSE score ≥ 20 (primary school) or ≥ 24 (junior school or above); and the ability to perform all activities of daily living independently [32]. The HCs had no history of neuropsychiatric disorders and no cognitive complaints.

The exclusion criteria for participants in this study included the following items: (1) left-handedness; (2) < 5 years of education; (3) the presence of any infarct with a diameter > 20 mm on T1-weighted images, cortical infarcts, or cerebral hemorrhage; (4) WMH mimics (e.g., multiple sclerosis, hypoxic-ischemic encephalopathy, and leukodystrophy); (5) a history of Parkinson's disease, epilepsy or psychiatric diseases; (6) use of medications that may affect cognitive function; (7) insufficient cooperation with the study procedures for any reason; and (8) a Hamilton depression scale score (24 items) ≥ 20. Sixty-eight WMH patients and 56 HCs with completed neuropsychological tests and MR scans fulfilled the above criteria. Two WMH participants with excessive head motion (>3 mm translation or >3° angular rotation on any axis) during scanning and one HC without a complete set of MRI data due to technical problems were excluded.

Based on cognitive ability, we then divided the WMH patients into two groups: (1) the WMH-MCI group, which included patients with a score ≥ 0.5 for at least one of the CDR domains, and objective evidence that one or more cognitive domains were impaired (age- and education-adjusted z-scores at least 1.5 SD below those of the HCs on one or more cognitive domains) [28, 32]; and (2) the WMH-nCI group, which included patients with a score of 0 on all six CDR domains, an MMSE score ≥ 24 (primary school) or ≥ 27 (junior school or above) and no objective evidence of impairment in any of the three cognitive domains. No participants were excluded by this procedure. Finally, a total of 121 participants (23 WMH-MCI, 43 WMH-nCI, and 55 HC) were included in the in-house dataset.

### MRI Acquisition

MR images were acquired on a 3.0T MR scanner (Discovery MR750, GE Healthcare, Milwaukee, WI, USA) using a 32-channel head array coil and included whole-brain T1-weighted, T2-weighted, fluid-attenuated inversion recovery (FLAIR), and a BRAin Volume (BRAVO) sequence. FLAIR images were collected using a repetition time (TR) = 8,000 ms, echo time (TE) = 160 ms, inversion time (TI) = 2,100 ms, flip angle (FA) = 111°, slice thickness = 5.0 mm, slice gap = 1.5 mm, data matrix = 512 × 512, and field of view (FOV) = 240 × 240 mm^2^. High-resolution anatomical T1-weighted images were obtained using a sagittal BRAVO sequence with TR = 8.16 ms, TE = 3.18 ms, TI = 50 ms, FA = 12°, number of slices = 188, slice thickness = 1.0 mm, data matrix = 256 × 256, FOV = 256 × 256 mm^2^, and voxel size = 1 mm × 1 mm × 1 mm.

### MRI Data Processing

We used the CAT12 toolbox (Computational Anatomy Toolbox; http://www.neuro.uni-jena.de/cat) implemented in SPM12 (Statistical Parametric Mapping, Institute of Neurology, London, UK) for voxel-based morphometry (VBM) and surface-based morphometry (for cortical thickness) analysis of imaging data [33–35]. Before pre-processing, all images were visually checked for the same orientation. Then they were corrected for bias-field inhomogeneities, aligned with the International Consortium for Brain Mapping template, and normalized using the DARTEL algorithm. The images were then segmented into GM, WM, and cerebrospinal fluid according to the tissue probability map provided in the CAT12 toolbox with voxel size = 1 mm × 1 mm × 1 mm. Furthermore, the segmented GM was modulated by the nonlinear normalization parameters to correct for individual brain size. After the pre-processing pipeline was completed, all scans passed the automated quality check protocol included in the CAT12 toolbox. For VBM analysis, the modulated and warped GM was finally smoothed by convolution with an isotropic Gaussian kernel, 6-mm full-width at half-maximum (FWHM) and total intracranial volume (TIV) was also calculated.

Cortical thickness analysis was implemented by the fully automated projection-based thickness method in the CAT12 toolbox. The algorithms uses tissue segmentation to estimate WN distance and project the local maxima to other GM voxels using the neighbor relationship described by the WM distance [33]. The surface data were resampled to the Human Connectome Project 32K template and were smoothed with a 15-mm FWHM Gaussian kernel.

As in our previous study [25], two independent raters (H.H. and Q.M.), blinded to the clinical information, manually segmented WMH from FLAIR images using ITK-SNAP 3.6.0 (http://www.itksnap.org/pmwiki/) and calculated the WMH volume as the sum of the WMH of all the image layers (details described in our previous study).

## Statistical Analyses

### Atlas-Based Analysis

To characterize the whole-brain GMV and cortical thickness changes of each participant, we used the Human Brainnetome Atlas [24], which consists of 246 subregions in the cerebrum (210 cortical and 36 subcortical) (http://atlas.brainnetome.org). All 246 regions for GMV and 210 regions for cortical thickness were investigated. One-way analysis of variance (ANOVA) and post-hoc analysis were implemented to identify differences in GMV and cortical thickness among the three groups (*P* < 0.05, Bonferroni corrected), controlling for age, gender, years of education, and TIV (for GMV) [36, 37] or age, gender, and years of education (for cortical thickness) [20] as confounding variables.

### Correlation Analyses and Mediation Analyses

Differences in clinical characteristics, correlation analysis, and mediation analysis were calculated using SPSS 20.0 (IBM Corp., Armonk, NY, USA). Pearson's correlation analyses were used to evaluate the relationships between adjusted GM measures that were controlled for age, gender, and years of education (for GMV, added controlled for TIV) in all identified regions using the atlas-based approach and the z-scores of each cognitive domain in the WMH-MCI group (*P* < 0.05). Furthermore, to determine whether the relationship between WMH and early cognitive decline can be explained by regional GM changes in the WMH patients, exploratory mediation analyses were further constructed in these patients using the PROCESS macro in SPSS [38], with altered GM measures (cortical thickness and GMV) of identified regions by between-group analysis as mediators and cognitive z-scores as outcomes, controlling for age, sex, years of education, and TIV. To improve the normality of the WMH variable, the total WMH volume of each individual was log-transformed. The relationship between WMH volume and cognitive performance (total effect, path c), and the associations between WMH volume and GM measures (path a), were also determined in each mediation model. Mediation analyses were evaluated using a bootstrap method (*n* = 5,000), and significant indirect effects were defined by a 95% confidence interval entirely above or below 0.

## Control Analyses

### Voxel-Based Analysis

To verify the results of GM abnormalities in WMH patients with and without cognitive impairment, complementary whole-brain voxel-wise analyses were also conducted among the three groups. The significant voxel-wise cortical thickness or GMV was determined using one-way ANOVA of the preprocessed imaging data among the three groups, with age, gender, years of education (adding TIV for GMV) as confounding variables. All analyses were restricted to the Brainnetome Atlas mask as the reference template, and the results were assessed at a peak threshold of *P* < 0.01 (FDR corrected, cluster size > 70 voxels).

### Replication of Main Results Using the ADNI Database

To further validate the robustness of the main results, we also compared the differences in cortical thickness and GMV among the three groups in the replication dataset from the ADNI (ADNI-2 and ADNI-GO) cohort with 154 individuals: 62 WMH-MCI, 23 WMH-nCI, and 69 HCs (http://adni.loni.usc.edu; details in the supplementary material). The same quality-control criteria and pre-processing pipeline described above were applied to the replication dataset. The WMH quantification approach in the replication dataset is detailed on the ADNI site (http://adni.loni.usc.edu) and in previous studies [39, 40]. To avoid the effect of correction for multiple comparisons and to evaluate the trend in whole-brain GM alterations among the three groups, correlation analyses between the in-house dataset and the replication dataset were calculated for each GM measure to explore whether the pattern of GM between-group differences was regionally consistent between the two datasets. For each GM measure, Pearson’s correlation of F-scores of ANOVA between the two datasets was calculated at the regional level and the voxel level.

### Replication of Main Results from Different Perspectives

To further verify the reproducibility of our main results, we repeated the between-group comparisons using different fine-grained brain atlases, including an atlas from the Human Connectome Project which consists of 360 regions [41], and two atlases of the Multiresolution Intrinsic Segmentation Template which consist of 325 regions and 444 regions [42]. We also repeated the analyses by adding hypertension and diabetes as covariates to investigate the potential confusion of the two risk factors which are considered to be involved in the progression of WMH underlying cognitive decline in WMH patients and verify the stability of our main results. In addition to the univariate analyses, we further conducted a group classification using logistic regression to explore whether the regional GM measures can be used as neuroimaging markers to discriminate WMH patients with and without early cognitive impairment.

## Results

### Participant Characteristics

The demographic, neuroimaging, and cognitive characteristics are the same as our previous study [25] and are summarized in Table 1. Briefly, no significant differences were found in age, gender, education level, and TIV among the three groups. Both WMH-MCI and WMH-nCI patients exhibited higher WMH volumes and Fazekas scores than HCs, whereas the differences of WMH volume and Fazekas score were not significant between the WMH-MCI and WMH-nCI groups. The WMH-MCI patients showed significantly worse cognitive performance (MMSE and z-score of the three cognitive domains) than the other two groups, and there were no differences in cognitive function between the WMH-nCI and HC groups (details of raw scores in each neuropsychological test in Table S1).

**Table 1:** Demographic and clinical features of the participants in the in-house dataset.

	HC (*n* = 55)	WMH-nCI (*n* = 43)	WMH-MCI (*n* = 23)	Overall *P* Value
*Demographic data*				
Age (years)	63.29 ± 6.50	65.72 ± 5.94	65.17 ± 6.65	0.089^a^
Gender (M/F)	32/23	27/16	15/8	0.813^b^
Education (years)	10.75 ± 3.29	10.28 ± 3.60	9.34 ± 3.89	0.251^a^
Hypertension, *n* (%)	10 (18.18%)	31 (72.09%)^d^	15 (62.22%)e	< 0.001^b^
Diabetes, *n* (%)	3 (5.45%)	7 (16.28%)^d^	5 (21.74%)e	< 0.001^b^
*Structural MRI features*				
TIV (cm^3^)	1517.73 ± 122.57	1550.85 ± 129.13	1500.78 ± 129.27	0.248^c^
Fazekas WMH score	1.00 ± 0.79	4.47 ± 1.18d	5.13 ± 1.10e	< 0.001^a^
Total WMH volume (cm^3^)	0.04 ± 0.09	13.53 ± 13.97^d^	18.19 ± 15.02e	< 0.001^a^
*Cognitive performance*				
MMSE	28.93 ± 1.30	28.77 ± 1.23	25.30 ± 2.72^e,f^	< 0.001^a^
Processing speed^g^	0.00 ± 0.91	0.04 ± 0.82	− 1.53 ± 0.95^e,f^	< 0.001^c^
Executive function^g^	0.00 ± 0.72	− 0.26 ± 0.57	− 1.52 ± 0.68^e,f^	< 0.001^c^
Memory^g^	0.00 ± 0.85	− 0.25 ± 0.87	− 1.71 ± 0.63^e,f^	< 0.001^c^

The data presented here are described in our previous study (Zhu et.al. 2019)

*TIV* total intracranial volume, *MMSE* mini-mental state examination

^a^Kruskal–Wallis test

^b^*χ*^2^ test

^c^One-way analysis of variance

^d^Significant difference between the WMH-nCI and HC groups

^e^Significant difference between the WMH-MCI and HC groups

^f^Significant difference between the WMH-MCI and WMH-nCI groups

^g^Z-scores. The performance of processing speed, executive function, and memory is presented as the compound z-scores for the related tests with HC scores as reference

### GM Alteration Patterns in WMH-MCI and WMH-nCI Patients

**Fig. 1:**
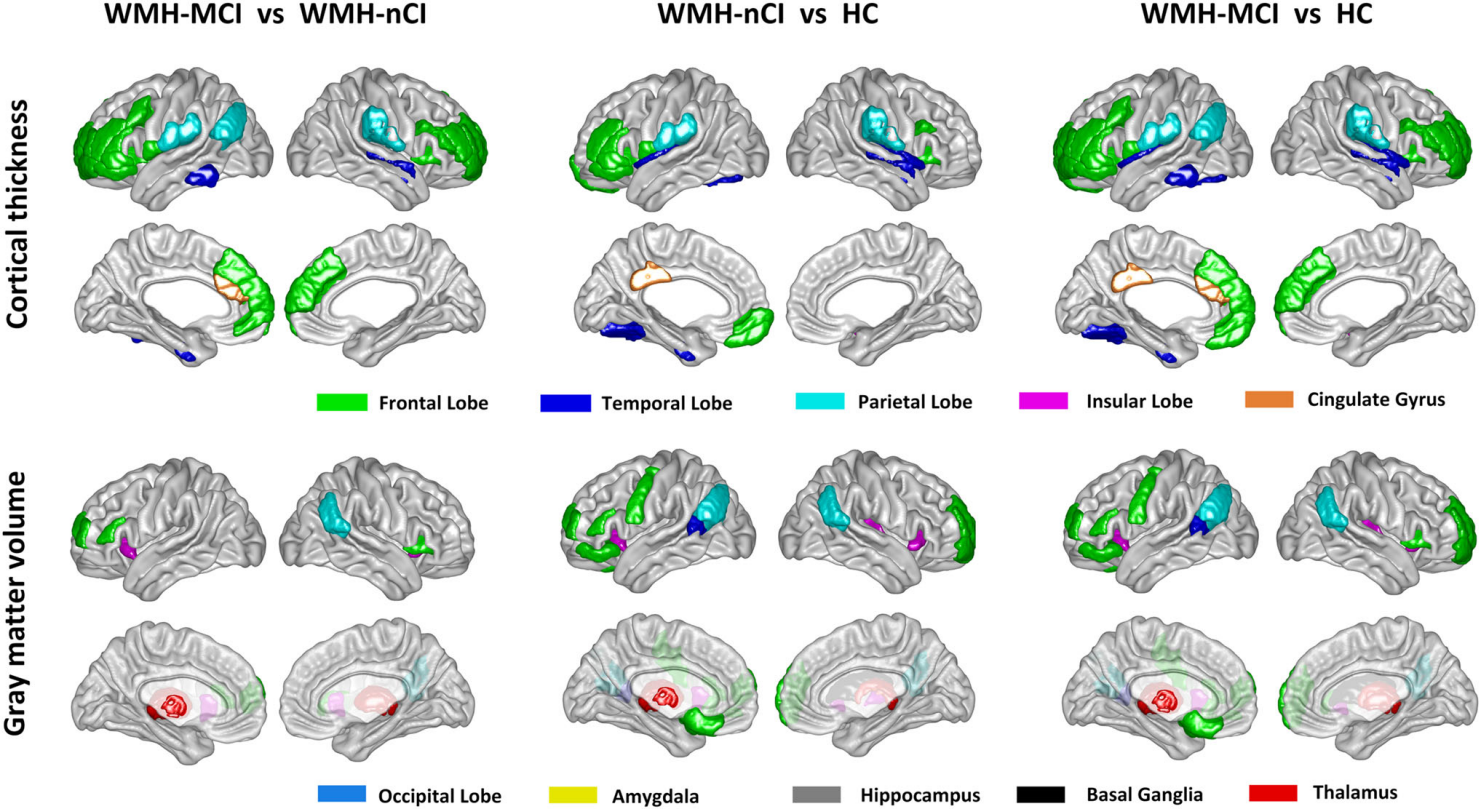
Differences in grey matter measures (grey matter volume and cortical thickness) among the three groups in the in-house dataset based on the Human Brainnetome atlas with Bonferroni correction (*P* <0.05). The regions of altered cortical thickness or grey matter volume across the three groups are represented by different colors.

At the regional level, significant GM alterations were distributed widely in cortical and subcortical regions among the three groups. Compared with HCs, both the WMH-MCI and the WMH-nCI patients showed a clear cortical GM atrophy pattern (the cortical thickness and GMV exhibited similar atrophy patterns) with predominant involvement of frontal, insular, parietal, and a few temporal regions, especially in the bilateral fronto-operculum/anterior insula, dorsolateral prefrontal cortex (DLPFC), and several regions of the inferior parietal lobe (IPL). More extensive and severe GM atrophy in these regions were identified in the WMH-MCI group relative to the WMH-nCI group. Subcortical nuclei were also affected in the WMH-MCI and the WMH-nCI patients. We found a significant reduction of GMV in the bilateral thalamus in the WMH-nCI patients compared with HCs, and nearly all subregions of the bilateral thalamus were more deeply involved in the WMH-MCI patients. Interestingly, relative to the HC group, the two WMH groups exhibited increased GMV in the bilateral caudate whereas no GM differences were identified between the WMH-MCI and WMH-nCI groups (Fig. 1; Tables S2, S3).

### Relationships Between GM Alteration, WMH, and Cognitive Performance

**Fig. 2:**
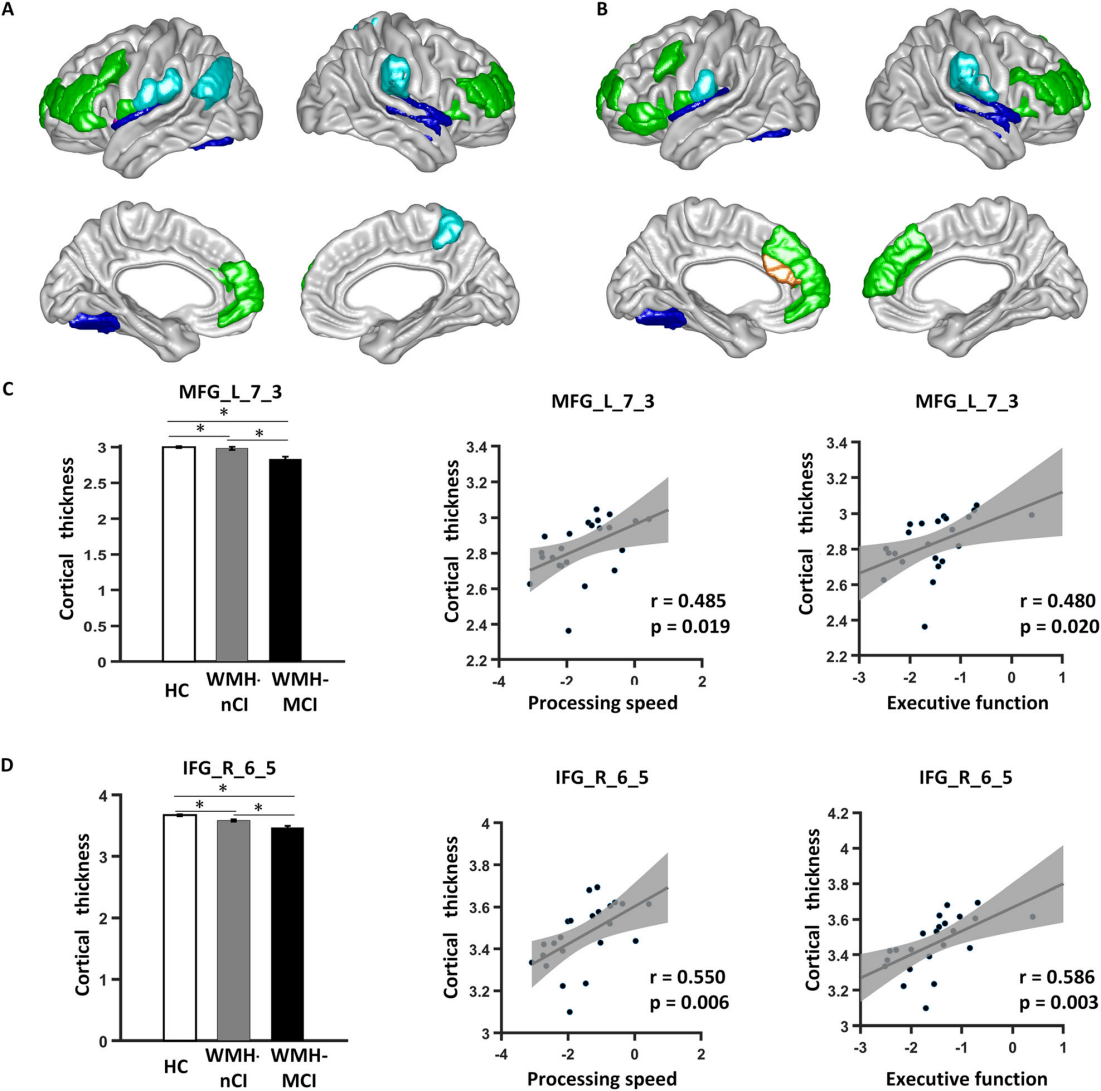
**A**, **B** Regions of significant correlation between mean cortical thickness and cognitive performance [processing speed (**A**) and executive function (**B**)] in WMH-MCI patients (*P* <0.05, uncorrected). C, D Mean cortical thickness in representative regions of the fronto-operculum (**C**) and DLPFC (**D**) in the three groups and scatter plots of the association between the mean cortical thickness in these regions and cognitive performance in WMH-MCI patients. DLPFC, dorsolateral prefrontal cortex; IFG, inferior frontal gyrus; MFG, middle frontal gyrus; L, left; R, right. *P < 0.05 (Bonferroni corrected).

**Fig. 3:**
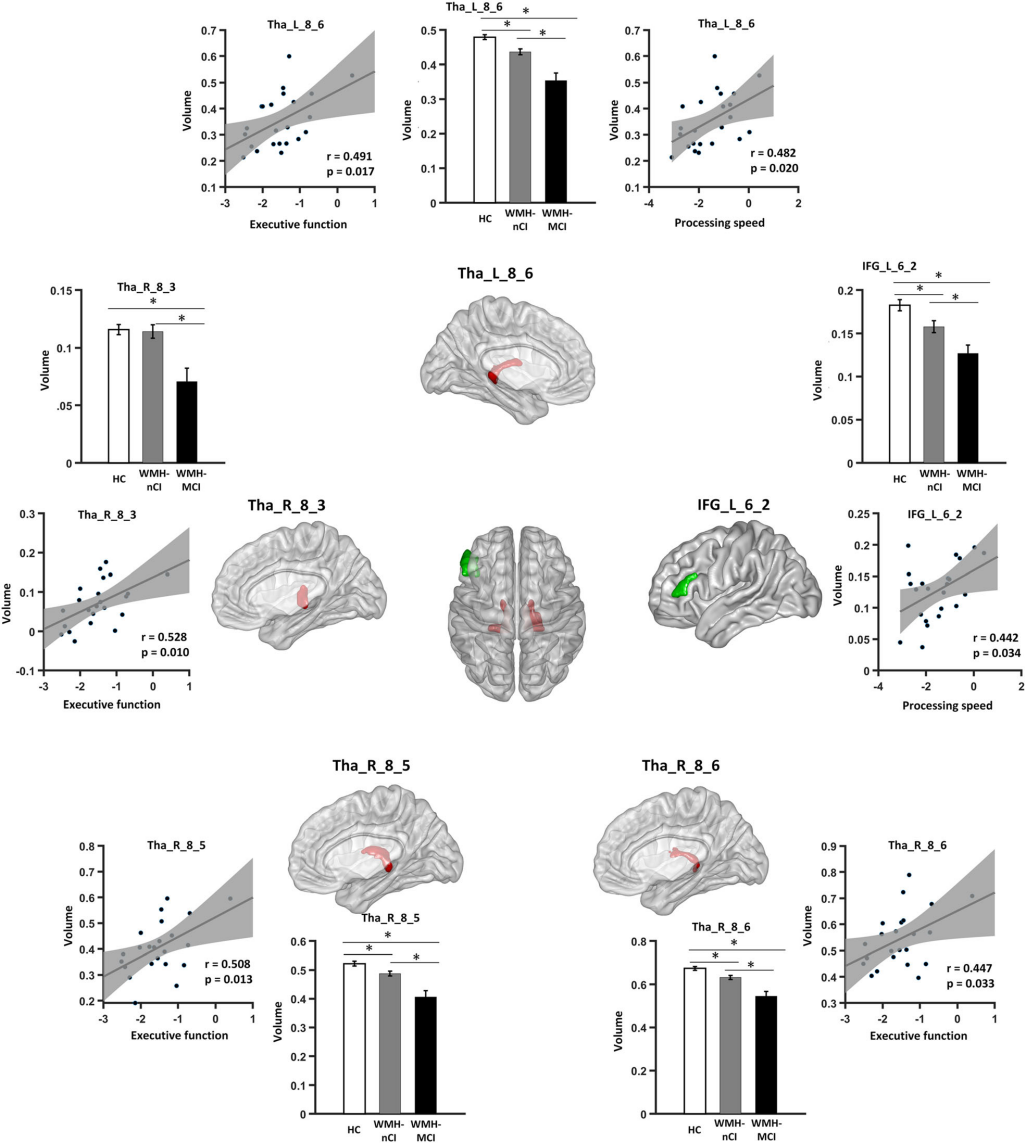
Regions of significant correlation between mean grey matter volume and cognitive performance in WMH-MCI patients. Bar graphs: group differences in mean grey matter volume of the identified regions. Scatter plots: correlations between the mean grey matter volume of identified regions and cognitive performance. IFG, inferior frontal gyrus; Tha, thalamus; L, left; R, right. *P < 0.05 (Bonferroni corrected)

In the WMH-MCI group, cortical thinning in most of the identified regions, mainly the bilateral fronto-operculum/anterior insula, DLPFC, and IPL, were positively associated with processing speed and executive function (Fig. 2 and Supplementary Table S4). Meanwhile, we identified GMV–cognition relationships in several subregions of the thalamus (for processing speed and executive function), as well as a region in the left fronto-operculum (for processing speed) (Fig. 3 and Table S5).

**Fig. 4:**
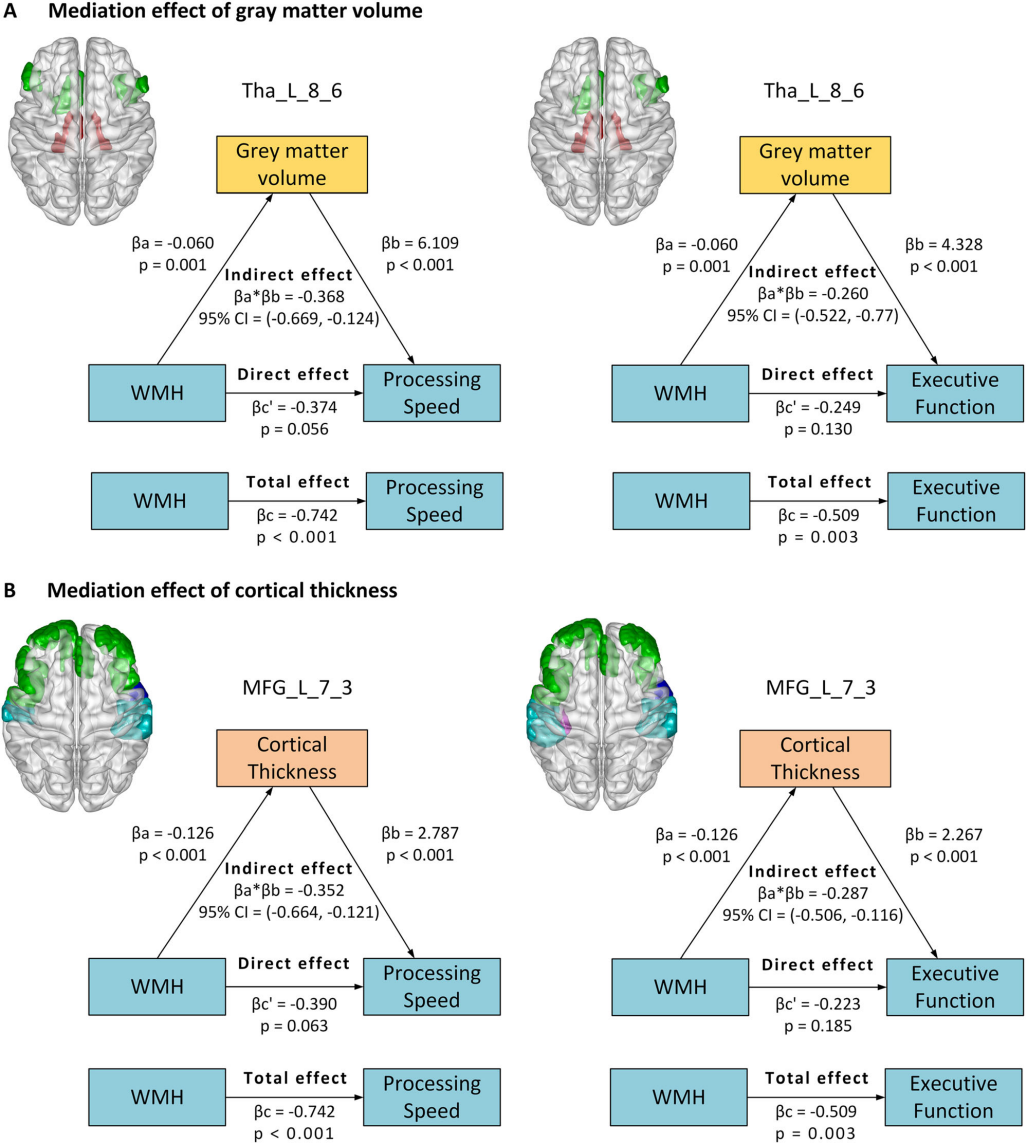
Mediation effect of grey matter measures between WMH and cognitive function. Diagrams show areas of grey matter volume (**A**) or cortical thickness (**B**) mediating the relationship between WMH volume and cognitive performance (processing speed or executive function), and mediation models of the pathways from WMH volume to grey matter atrophy for grey matter volume (**A**) and cortical thickness (**B**) in representative regions (two subregions in the thalamus and frontal cortex) to reduce cognitive performance. In each model, age, sex, years of education, and TIV were entered as covariates. βa and βb represent the coefficients of the relationships between WMH volume and the regional grey matter measures (grey matter volume or cortical thickness), and the associations between the grey matter measures and cognition (when both WMH volume and grey matter measures were entered into the model as predicting variables). The mediating role of regional grey matter atrophy on the association between WMH and cognition is defined when the 95% confidence interval is entirely above or below 0 for 5,000 bootstrapping iterations.

In all WMH patients, there were significant associations between log-transformed WMH volume and processing speed (*r* = –0.742, *P* < 0.001) and executive function (*r* = –0.509, *P* = 0.003), and no correlation between log-transformed WMH volume and memory (*r* = –0.311, *P* = 0.152). Significant associations were also found in the WMH patients between log-transformed WMH volume and GMV reduction in the thalamus, as well as reduction of GMV and cortical thickness in multiple identified frontal, insular, parietal, and a few temporal regions. Since no significant correlation was evaluated between WMH load and memory, mediation models were established only for processing speed and executive function. The mediation effects of WMH on cognition were identified *via* GM atrophy in several cortical and subcortical regions, mainly several regions of the thalamus (for GMV), and fronto-insular cortices (for cortical thickness and/or GMV). In addition, the mediation analyses indicated that specific GM atrophy mainly mediates the relationships between WMH and processing speed or executive function (Fig. 4 and Tables S6, S7).

### Control Results

**Fig. 5:**
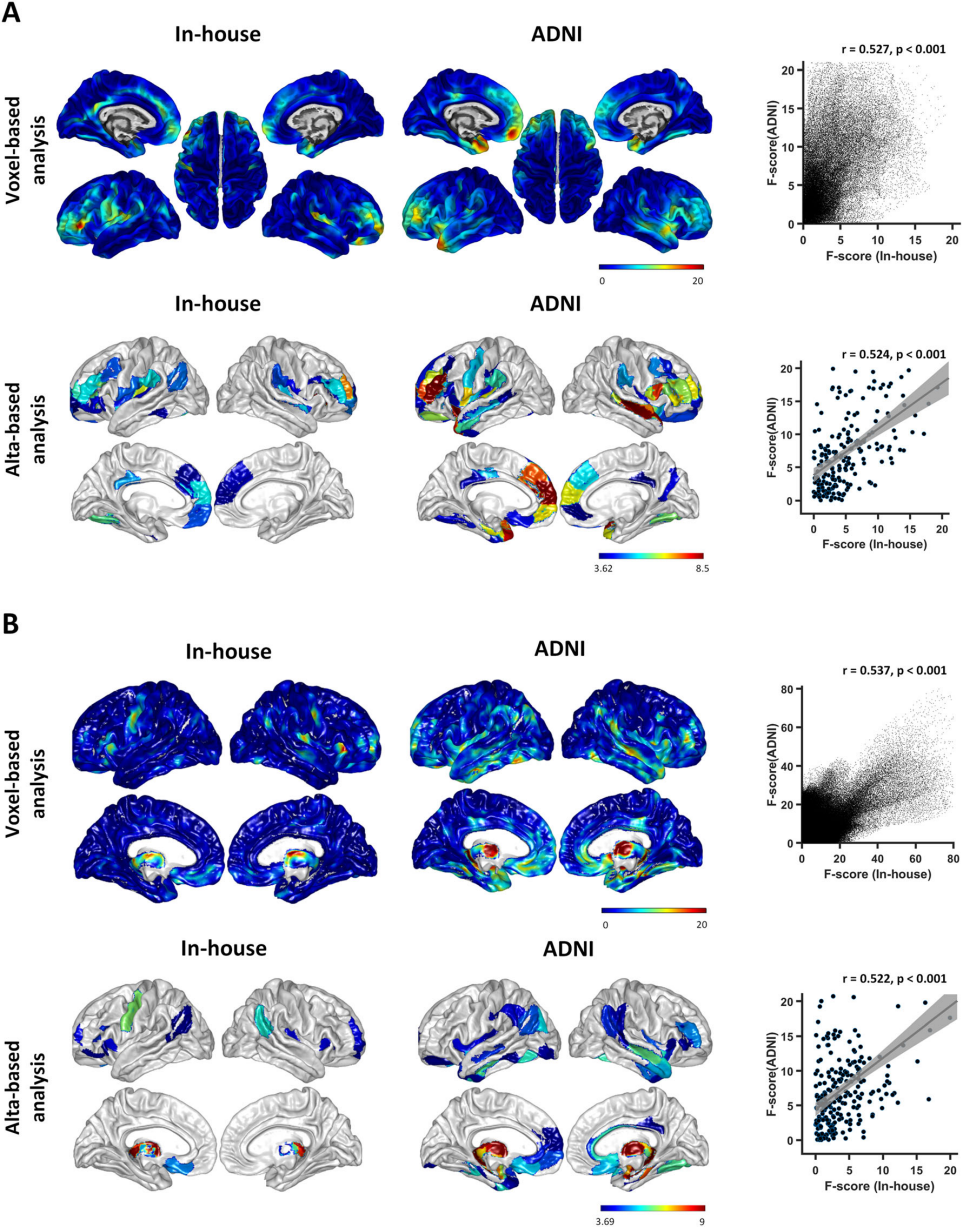
Correlation analyses of the among-group differences in grey matter measures between the in-house dataset and the replication dataset from the ADNI database. Diagrams showing the statistical maps (left) and scatter plots (right) of correlation analyses between the *F* scores of ANOVA for cortical thickness (**A**) and grey matter volume (**B**) of the two independent datasets at the voxel and regional levels. The warmer and cooler colors indicate higher and lower correlation of grey matter alterations between the two datasets. ADNI, the Alzheimer's Disease Neuroimaging Initiative.

At the voxel level, the similar GM alteration maps (identified by GMV and cortical thickness) among the three groups were well replicated in the replication dataset from the ADNI database (details in Figs S1, S2 and Tables S8, S9). Briefly, the replication dataset from the ADNI database showed a very similar alteration patterns among the WMH-MCI, WMH-nCI, and HC groups in the demographic, neuroimaging, and cognitive metrics (Table S10). The patterns of GM abnormalities showed spatial consistency between the two datasets at either the regional level (cortical thickness, *r* = 0.524, *P* < 0.001; GMV, *r* = 0.522, *P* < 0.001) or the voxel level (cortical thickness, *r* = 0.537, *P* < 0.001; GMV, *r* = 0.527, *P* < 0.001) (Fig. 5). The WMH-MCI and WMH-nCI groups of the replication dataset exhibited GM atrophy mainly in the bilateral thalamus, fronto-insular, temporal, and a few parietal regions compared with the HC group, and GM atrophy was more widespread and severe in the WMH-MCI group (Fig. S3 and Tables S11, S12).

As a control analysis for the choice of brain parcellation, we created a similar GM alteration map (revealed by the cortical thickness and GMV) among the three groups using the different brain atlases (Figs. S4, S5). Furthermore, we found significant correlations (*r* = 0.95, *P* < 0.0001 for cortical thickness; *r* = 0.97, *P* < 0.0001 for GMV) between the F-score in the main result with and without controlling for hypertension and diabetes, emphasizing the robustness of our results and indicating that other risk factors such as hypertension and diabetes are unlikely to provide a plausible explanation of the GM alterations underlying cognitive decline in WMH patients revealed in this study. In addition, the exploratory group classifications showed that regional GM measures can separate the WMH-MCI and WMH-nCI groups with relatively high accuracy (77.3% for cortical thickness; 83.3% for GMV; details in Tables S13, S14).

## Discussion

In this work, we systemically investigated differences in whole-brain GM maps (GMV and cortical thickness) among moderate to severe WMH patients with and without MCI, and HCs at both the regional and the voxel level. Compared with HCs, both the WMH-MCI group and the WMH-nCI group showed a similar GM atrophy pattern in the bilateral thalamus, fronto-insular cortices, and a few parietal-temporal regions, and the pattern was more extensive and severe in the WMH-MCI than in the WMH-nCI group. Importantly, the GM–cognition correlation analyses and mediation analyses among GM, WMH, and cognition further identified the crucial role of specific GM atrophy in the thalamus and fronto-insular cortices underlying the early cognitive decline in WMH patients. Furthermore, the main results were convincingly well replicated in a series of control analyses including analyses at the voxel level, in an independent dataset from the ADNI database, and using different templates. In addition, exploratory classification analyses indicated that regional GM measures also distinguished WMH patients with and without early cognitive impairment. Altogether, these findings provide a novel insight into the mechanisms underlying WMH and subsequent cognitive decline.

Compared to HCs, both of the WMH groups exhibited volumetric loss of deep subcortical GM nuclei, especially the bilateral thalamus, in line with previous studies focused on subcortical vascular MCI patients [12] or patients with high WMH load from a community-dwelling cohort [43]. Furthermore, to extend their findings, we found that the WMH-MCI patients showed a more widespread and severe reduction of thalamic GMV than the WMH-nCI patients and further identified positive correlations between several subregions of the thalamus and cognition (processing speed and executive function) within the WMH-MCI group. Notably, our study for the first time revealed a mediating role of thalamic atrophy on the relationship between WMH load and cognitive decline in patients with WMH. Robust evidence has suggested that the thalamus works as a relay hub to the cerebral cortex and contributes to cognitive processing in diverse domains [44–46]. The characteristic atrophy of the thalamus underlying cognitive decline in WMH patients may be explained by two mechanisms. Primarily, as a subcortical manifestation that can be partly attributed to the chronic ischemia of CSVD, WMH not only reflects damage of the WM but also can invade subcortical GM nuclei directly [47]. The other explanation is that the thalamus has dense structural and functional connections to cortical regions, which are therefore vulnerable to hypometabolism and degeneration due to disruption of WM tracts even if there is no visible WMH in the thalamus on MRI [48–51]. In our previous studies, we have demonstrated functional thalamic disconnections in the cognitive decline of WMH patients using resting-state functional MRI [25]. And DTI studies have also shown that some projection fibers connecting the thalamus to cortical regions (i.e., the anterior thalamic radiation) are correlated with cognitive decline in CSVD patients with a high WMH load [52, 53]. Given of the above, there is convergent evidence that the thalamus may be susceptible to WMH progression and damage of the thalamus develops along with the course of cognitive decline in WMH patients.

For the cerebral cortex, our results revealed shared and specific GM alterations in the WMH-MCI group relative to the WMH-nCI group and HCs; these mainly included reduced GMV and thinner cortical thickness in the fronto-insular cortices and a few regions of the parietal (mainly IPL) and temporal cortices. In particular, the cortical atrophy (mainly cortical thinning) in the majority of these regions was associated with cognitive decline in the WMH-MCI group. Of note, previous studies have explored the pattern of cortical atrophy in patients with WMH and MCI [10–13]. Although it was hampered by the use of heterogeneous patient groups and methodological differences, our study, consistent with prior studies, showed that the reduction of cortical thickness and GMV in fronto-insular cortices was identified in patients with MCI and a high WMH load. The fronto-insular cortices, together with the thalamus, constitute the core regions of the salience network (SN), and are involved in a wide range of cognitive processes [54–56]. Meanwhile, the DLPFC and IPL, which are the hub of the executive control network (ECN) [56], were also identified as the characteristic atrophic regions in WMH-MCI patients compared with WMH-nCI patients and HCs. Playing essential and dissociable roles in human emotion and cognition [56, 57], the structural alterations of the SN and ECN coincide with the functional disconnection patterns underlying cognitive impairment in WMH patients revealed by previous studies [25, 58, 59]. Hence, we speculate that the selective GM atrophy of the hub regions in the SN and ECN may lead to functional abnormalities across the cognitive networks and this could be the crucial mechanism of WMH-related cognitive impairment.

One of the distinctive aspects in the present study was that we compared GM alterations among WMH patients with MCI, with those whose cognition was preserved and HCs. Thus, we obtained the characteristic GM alteration pattern in clinically early-stage WMH patients. Interestingly, compared with HCs, GM atrophy was also observed in the WMH-nCI patients, particularly in the thalamus and fronto-insular cortices noted above. In addition to being hub regions in cognitive networks, these regions that constitute the fronto-subcortical circuit are also thought to be involved in emotion and motor control. Thus, the specific GM atrophy of the thalamus and fronto-insular cortices in the WMH-nCI group corresponded to some extent to the disturbance of emotion and gait in WMH patients usually with an insidious onset that may occur before the cognitive impairment [1, 60]. Another interesting finding is that increased GMV of the bilateral caudate was detected in both the WMH-MCI and WMH-nCI patients compared with HCs, consistent with a previous study [11]. These novel findings may be a compensatory mechanism in the clinically early stage of WMH. Another possible explanation is that disturbance of the caudate may be responsible for the potential mood and motor disturbance in WMH patients, as revealed in 22q11.2 deletion syndrome [61] and vascular parkinsonism [62]. Although these findings were obtained at both the regional and the voxel level, and were repeated in the replication dataset from the ADNI database, further investigations is needed to verify the generalization and explore the mechanisms.

We further evaluated the relationships among GM alteration, WMH, and cognition in WMH patients. To date, although some evidence has been obtained, the pathophysiology of cortical atrophy in WMH and subsequent cognitive impairment remains elusive. As noted in prior studies [16, 63], there are two possible mechanisms to explain this issue. First, considered to be the most important imaging manifestations and reflect the overall burden of CSVD, WMH may disturb WM fibers connecting cortical to subcortical GM, leading to secondary damage of the axonal cytoskeleton and cortical degeneration [64–66]. Our results add to the growing body of literature that implicates the specific disruption of WM tracts (i.e., the anterior thalamic radiation and corpus callosum) connected between subcortical and cortical regions in WMH patients [53, 65], which may cause a characteristic structurally and functionally aberrant fronto-subcortical network and eventually lead to cognitive impairment. The other possible mechanism is that direct damage by CSVD, such as cortical microinfarcts and microbleeds, may also be responsible for cortical atrophy in WMH and the subsequent cognitive impairment. Nevertheless, it is difficult to explain the characteristic damage pattern in WMH patients with this hypothesis and further investigation is needed in studies with susceptibility-weighted images, 3D FLAIR, or double inversion recovery sequences [67, 68].

We also performed a series of control analyses (repeated the between-group comparisons at the voxel level, in an independent dataset from the ADNI database, using multiple measures and different atlases, and further controlling for risk factors such as hypertension and diabetes) to verify the reproducibility and generalization of the main results of GM alterations in WMH patients with and without cognitive impairment. Notably, whether revealed in another independent dataset or using different templates, the GM alterations across the three groups exhibited very similar distribution patterns, highlighting the important role of the thalamus and fronto-insular cortices in WMH progression and subsequent cognitive impairment. Furthermore, since different sensitivity in capturing different types of structural changes and pathological processes [23, 69, 70], the similar GM alteration patterns evaluated among the three groups by using different measures (GMV *vs* cortical thickness, and atlas-based *vs* voxel-based approaches), were able to demonstrate the reproducibility and generalization of our main results from multiple perspectives, although there were great disparities in age, race, and ethnicity, as well as MRI scanners between two datasets which may lead to differences of altered regions across the three groups revealed by the two datasets. Besides, the results of exploratory classification analyses indicated that regional GM measures may serve as a novel and potential marker for detecting the early cognitive decline in patients with a high WMH load at the individual level.

Despite these advances, this study had several limitations that should be addressed. First, the sample size of the in-house dataset was relatively small, particularly for the WMH-MCI group, and the sizes of the WMH-MCI and WMH-nCI groups were unbalanced due to the study design, which may lead to lower statistical power. Based on studies focused on the neuroimaging features of AD [71, 72], the characteristic GM alteration pattern in WMH patients with MCI needs further cross-validation by large-scale multi-center studies in the future. Second, we did not conduct a comprehensive evaluation of motor and mood disturbances which are common in patients with WMH. Hence, an influence of these factors on the results cannot be completely excluded. Finally, since various types of pathology (cerebral ischemia, aging, and AD-related degeneration) may participate in the mechanisms of WMH progression and cognitive impairment in WMH patients [73], longitudinal studies with Aβ and tau measures are warranted, to reach further consensus on specific GM alterations underlying cognitive decline in WMH and evaluate the clinical value of GM for the prediction of early cognitive impairment in WMH patients.

## Conclusion

Here, we demonstrated the characteristic structural GM alteration pattern in WMH patients with and without MCI, and further indicated that specific GM atrophy in the thalamus and fronto-insular cortices underlie the cognitive decline in WMH patients. Importantly, the alteration pattern in the main results was verified in a series of control analyses, highlighting the robustness and generalizability of our data. Our results provide novel insights into the mechanisms of WMH progression and the subsequent cognitive impairment in patients with a high WMH load.

## Supplementary Information

Below is the link to the electronic supplementary material.

Supplementary file1 (PDF 1409 KB)
