# Supplementary material for: Cortical and Subcortical Grey Matter Abnormalities in White Matter Hyperintensities and Subsequent Cognitive Impairment
Source: Neurosci Bull. 2021 Apr 7;37(6):789–803. doi: 10.1007/s12264-021-00657-0 (PMC8192646; doi:10.1007/s12264-021-00657-0)
Supplement: Supplementary file 1 — Supplementary file1 (PDF 1409 KB) [file 12264_2021_657_MOESM1_ESM.pdf]

Supplementary Information

Supplementary Material and Methods

Assessment of cognitive performance

The participants underwent clinical interviews and a battery of neuropsychological tests, including the Mini-Mental State Examination (MMSE), the Clinical Dementia Rating, the Trail Making Test (TMT), the Symbol Digit Modalities Test (SDMT), the Digit Span Test (DST), the Verbal Fluency Test (VFT), the Auditory Verbal Learning Test (AVLT), and the Hamilton depression rating scale (24-items). For comparisons of performance across the tests, we calculated the z-scores (individual test scores – mean scores of HCs) / standard deviation of HCs) for each test[1]. In particular, as higher TMT scores indicate worse cognitive performance, we multiplied the individual z-scores for these tests by –1[1]. To represent the cognitive abilities more robustly and to reduce random errors, the neuropsychological tests were composited into three cognitive domains[2]: processing speed (TMT-A and SDMT)[3], executive function (DST backward, VFT and TMT-B)[4], and memory (AVLT immediate recall, short delay recall, long delay recall, and long delay recognition)[5]. The performance of the participants on tests encompassing each cognitive domain was presented as the compound z-score calculated by averaging z-scores for the corresponding tests[1, 6].

Participants in the replication dataset

To further validate the robustness of the main results, an independent dataset from the ADNI (ADNI-2 and ADNI-GO) database was also used. All participants provided written informed consent.

Additional information about the ADNI dataset can be found at http://adni.loni.usc.edu/wp-content/uploads/how_to_apply/ADNI_Acknowledgement_List.pdf. We restricted the analyses to MCI [including early MCI (EMCI) and late MCI (LMCI)] and cognitively normal (CN) individuals whose data of 3D T1WI, T2 FLAIR, WMH volume, and the number of infarcts were available. Details of inclusion and exclusion criteria for EMCI, LMCI, and CN were as described previously[7]. Considering the high heterogeneity of data from different MR manufacturers, we further restricted analyses to participants whose MR data were collected on Siemens MR scanners (since >60% of individuals met the above criteria underwent MR scans on the Siemens MR scanner). After evaluating the extents of WMH visually (by Fazekas scale), we further defined MCI patients with moderate to severe MCI as the WMH-MCI group and excluded the MCI patients without moderate to severe WMH. Meanwhile, we divided the CN participants into two group: (1) the WMH-nCI group (participants with moderate to severe WMH); (2) the HC group (CN subjects with no or mild WMH). In addition, WMH-MCI and WMH-nCI patients with cortical infarcts, or any infarct with a diameter >20 mm on T1-weighted images were excluded, and no infarcts were detected in HCs. Finally, the replication dataset recruited 154 individuals: 62 with MCI and moderate to severe WMH (WMH-MCI), 23 with WMH-nCI, and 69 HCs.

Image acquisition protocol of the replication dataset

The protocol of data acquisition of the replication dataset included a FLAIR sequence and a T1-weighted 3D magnetization-prepared rapid gradient-echo imaging. A full description of the parameters used in the MRI examinations can be found at http://adni.loni.usc.edu

Supplementary Tables.

Table S1. Neuropsychological and head motion data.

	HC (n = 55)	WMH-nCI (n = 43)	WMH-MCI (n = 23)	Overall p Value
SDMT	27.33 ± 11.10	26.35 ± 10.72	13.61 ± 5.89[a,b]	< 0.001[c]
VFT	23.84 ± 7.29	22.81 ± 5.54	15.96 ± 5.34[a,b]	< 0.001[d]
TMT-A	75.01 ± 36.55	69.10 ± 31.80	141.91 ± 56.25[a,b]	< 0.001[c]
TMT-B	134.14 ± 55.95	146.53 ± 52.64	258.74 ± 83.21[a,b]	< 0.001[c]
DST backward	4.42 ± 1.20	3.93 ± 0.88	2.91 ± 0.79[a,b]	< 0.001[c]
AVLT immediate recall	19.78 ± 4.96	18.86 ± 4.74	11.78 ± 2.95[a,b]	< 0.001[c]
AVLT short delay recall	7.53 ± 2.12	6.91 ± 2.43	3.52 ± 1.44[a,b]	< 0.001[d]
AVLT long delay recall	7.16 ± 2.28	6.49 ± 2.44	2.74 ± 1.63[a,b]	< 0.001[d]
AVLT recognition	11.35 ± 0.91	11.16 ± 0.92	10.09 ± 1.08[a,b]	< 0.001[c]
mean motion	0.08 ± 0.08	0.08 ± 0.05	0.11 ± 0.08	0.131

The data are presented as the mean ± SD.

[a] Significant difference between the WMH-MCI and HC groups.

[b] Significant difference between the WMH-MCI and WMH-nCI groups.

[c] Kruskal-Wallis test.

[d] One-way analysis of variance.

AVLT, Auditory Verbal Learning Test; DST, Digital Span Test; SDMT, Symbol Digit Modalities Test; TMT, Trail Making Test; VFT, Verbal Fluency Test.

Table S2. Regional differences in cortical thickness among the WMH-MCI, WMH-nCI, and HC groups in the in-house dataset using the Human Brainnetome Atlas.

Brain Area	Mean cortical thickness			ANOVA		HC vs WMH-nCI		HC vs WMH-MCI		WMH-nCI vs WMH-	
	HC	WMH-nCI	WMH-MCI	*F*	*P*	*t*	*P*	*t*	*P*	*t*	*P*
SFG_L_7_7	3.358 ± 0.013	3.313 ± 0.020	3.202 ± 0.028	14.09247	< 0.00001	1.91548	0.05841	5.78819	< 0.00001	3.20344	0.00212
MFG_L_7_3	3.001 ± 0.014	2.982 ± 0.021	2.832 ± 0.034	14.79289	< 0.00001	0.79750	0.42713	5.48043	< 0.00001	3.97006	0.00018
MFG_R_7_3	3.103 ± 0.012	3.077 ± 0.018	2.935 ± 0.029	19.27336	< 0.00001	1.21656	0.22675	6.29174	< 0.00001	4.43914	0.00004
MFG_L_7_4	2.980 ± 0.012	2.939 ± 0.016	2.841 ± 0.030	13.99281	< 0.00001	2.13444	0.03535	5.23674	< 0.00001	3.18810	0.00222
MFG_R_7_4	3.047 ± 0.013	3.026 ± 0.018	2.910 ± 0.028	13.90096	< 0.00001	1.04039	0.30077	5.49915	< 0.00001	3.66752	0.00050
IFG_L_6_2	3.196 ± 0.015	3.141 ± 0.021	3.008 ± 0.035	15.79893	< 0.00001	2.15124	0.03397	5.76112	< 0.00001	3.42214	0.00109
IFG_L_6_5	3.582 ± 0.016	3.504 ± 0.020	3.373 ± 0.042	17.87998	< 0.00001	3.11430	0.00243	5.68081	< 0.00001	3.20562	0.00210
IFG_R_6_5	3.672 ± 0.015	3.584 ± 0.017	3.465 ± 0.032	24.37051	< 0.00001	3.85652	0.00021	6.66173	< 0.00001	3.56972	0.00068
STG_R_6_6	3.460 ± 0.019	3.375 ± 0.023	3.278 ± 0.030	13.64572	< 0.00001	2.92294	0.00432	5.16093	< 0.00001	2.55012	0.01317
FuG_L_3_2	2.925 ± 0.020	2.769 ± 0.024	2.769 ± 0.034	15.37175	< 0.00001	5.05513	<0.00001	4.15124	0.00009	0.00438	0.99652
IPL_L_6_6	3.109 ± 0.013	3.044 ± 0.020	2.957 ± 0.030	14.10483	< 0.00001	2.89810	0.00465	5.54765	< 0.00001	2.52513	0.01405
INS_L_6_1	3.695 ± 0.022	3.521 ± 0.028	3.428 ± 0.062	17.15477	< 0.00001	4.92684	<0.00001	5.10621	< 0.00001	1.56978	0.12140
INS_L_6_4	4.646 ± 0.037	4.420 ± 0.038	4.355 ± 0.054	14.02756	< 0.00001	4.23802	0.00005	4.35958	0.00004	0.98896	0.32641
MFG_R_7_7	3.028 ± 0.012	3.021 ± 0.014	2.910 ± 0.026	12.48493	0.00001	0.38040	0.70449	4.62995	0.00001	4.06214	0.00014
INS_R_6_1	3.595 ± 0.026	3.436 ± 0.033	3.299 ± 0.075	13.44767	0.00001	3.83399	0.00023	4.69011	0.00001	1.93120	0.05789
CG_L_7_1	2.769 ± 0.018	2.660 ± 0.029	2.584 ± 0.034	12.71890	0.00001	3.46817	0.00079	5.23363	< 0.00001	1.57911	0.11924
MFG_L_7_2	3.127 ± 0.014	3.108 ± 0.020	2.979 ± 0.034	11.81946	0.00002	0.81471	0.41726	4.82442	0.00001	3.52473	0.00079
OrG_L_6_1	3.153 ± 0.013	3.100 ± 0.020	3.016 ± 0.029	11.70222	0.00002	2.38289	0.01915	5.14834	< 0.00001	2.48637	0.01553
OrG_L_6_4	2.837 ± 0.015	2.732 ± 0.020	2.723 ± 0.030	11.69103	0.00002	4.38046	0.00003	3.88309	0.00022	0.23056	0.81840
PrG_L_6_5	3.351 ± 0.015	3.298 ± 0.019	3.208 ± 0.023	11.99506	0.00002	2.16679	0.03273	5.12767	< 0.00001	2.84998	0.00588
IPL_L_6_5	2.912 ± 0.016	2.869 ± 0.018	2.766 ± 0.029	12.20390	0.00002	1.87342	0.06405	4.83658	0.00001	3.22639	0.00198
IPL_R_6_6	3.110 ± 0.013	3.053 ± 0.018	2.980 ± 0.025	11.68184	0.00002	2.58214	0.01133	5.05694	< 0.00001	2.35226	0.02175
PoG_L_4_2	3.084 ± 0.016	3.008 ± 0.019	2.931 ± 0.035	12.02828	0.00002	3.12625	0.00234	4.59487	0.00002	2.13612	0.03650
INS_R_6_4	4.923 ± 0.035	4.700 ± 0.035	4.707 ± 0.061	11.27216	0.00003	4.46643	0.00002	3.24847	0.00173	-0.10756	0.91468
INS_L_6_5	3.452 ± 0.030	3.280 ± 0.040	3.208 ± 0.049	11.53828	0.00003	3.66361	0.00041	4.64744	0.00001	1.08161	0.28349

INS_L_6_6	3.895 ± 0.021	3.740 ± 0.036	3.707 ± 0.046	10.80116	0.00005	3.97976	0.00013	4.27208	0.00006	0.49898	0.61950
IFG_R_6_1	3.147 ± 0.020	3.080 ± 0.024	2.963 ± 0.043	10.53481	0.00006	2.18164	0.03158	4.49313	0.00002	2.59412	0.01174
IFG_L_6_4	2.992 ± 0.015	2.935 ± 0.020	2.858 ± 0.023	10.64510	0.00006	2.34326	0.02118	4.78583	0.00001	2.40743	0.01896
PoG_R_4_2	2.969 ± 0.015	2.903 ± 0.017	2.835 ± 0.030	10.60786	0.00006	2.83487	0.00559	4.32796	0.00005	2.08693	0.04088
STG_L_6_3	3.227 ± 0.020	3.121 ± 0.028	3.063 ± 0.030	10.42144	0.00007	3.20942	0.00181	4.52489	0.00002	1.31619	0.19281
OrG_L_6_6	3.259 ± 0.017	3.201 ± 0.018	3.121 ± 0.028	9.91380	0.00010	2.30248	0.02347	4.28367	0.00005	2.45218	0.01694
SFG_R_7_6	3.497 ± 0.015	3.485 ± 0.019	3.364 ± 0.032	9.90001	0.00011	0.49297	0.62316	4.32329	0.00005	3.47451	0.00092
CG_L_7_3	3.775 ± 0.024	3.715 ± 0.030	3.566 ± 0.044	9.81522	0.00011	1.58935	0.11527	4.48842	0.00003	2.89313	0.00521
PhG_L_6_1	5.001 ± 0.041	4.878 ± 0.037	4.635 ± 0.011	9.65259	0.00013	2.15285	0.03384	3.90283	0.00020	2.62203	0.01091
ITG_L_7_6	2.822 ± 0.032	2.841 ± 0.035	2.591 ± 0.053	9.56598	0.00014	-0.41233	0.68102	3.83005	0.00026	4.10646	0.00012
IFG_R_6_2	3.466 ± 0.017	3.436 ± 0.020	3.326 ± 0.028	9.44785	0.00016	1.12991	0.26133	4.38919	0.00004	3.19711	0.00216
STG_R_6_3	3.436 ± 0.020	3.349 ± 0.026	3.269 ± 0.035	9.41558	0.00016	2.66768	0.00897	4.26056	0.00006	1.84171	0.07015
SFG_R_7_7	3.344 ± 0.013	3.306 ± 0.021	3.211 ± 0.032	9.29397	0.00018	1.60507	0.11176	4.57999	0.00002	2.57892	0.01222
SFG_L_7_6	3.451 ± 0.013	3.414 ± 0.020	3.326 ± 0.030	9.24132	0.00019	1.66206	0.09976	4.54871	0.00002	2.55608	0.01297
MFG_L_7_7	3.109 ± 0.013	3.091 ± 0.019	2.985 ± 0.030	9.14325	0.00020	0.75728	0.45074	4.35901	0.00004	3.09546	0.00291

The cortical thickness of the three groups is shown as the mean ± SD (P <0.05, Bonferroni corrected). F, F value of ANOVA; t, t score of the *post hoc* analysis.

CG, cingulate gyrus; FuG, fusiform gyrus; IFG, inferior frontal gyrus; INS, insular gyrus; IPL, inferior parietal lobule; ITG, inferior temporal gyrus; MFG, middle frontal gyrus; PhG, parahippocampal gyrus; PoG, postcentral gyrus; PrG, precentral gyrus; SFG, superior frontal gyrus; STG, superior temporal gyrus; L, left; R, right. Detailed information on the brain regions is available at http://atlas.brainnetome.org/.

Table S3. Regional differences in grey matter volume among the WMH-MCI, WMH-nCI, and HC groups in the in-house dataset using the Human Brainnetome Atlas.

Brain Area	Mean GMV			ANOVA		HC vs WMH-nCI		HC vs WMH-MCI		WMH-nCI vs WMH-MCI	
	HC	WMH-nCI	WMH-MCI	*F*	*P*	*t*	*P*	*t*	*P*	*t*	*P*
PrG_L_6_1	0.185 ± 0.006	0.143 ± 0.008	0.121 ± 0.012	16.79386	< 0.00001	4.38074	0.00003	5.40064	< 0.00001	1.55692	0.12442
BG_R_6_5	0.506 ± 0.007	0.592 ± 0.013	0.579 ± 0.021	16.97439	< 0.00001	-	< 0.00001	-	0.00008	0.55837	0.57854
IPL_R_6_5	0.158 ± 0.004	0.140 ± 0.005	0.119 ± 0.006	15.12515	< 0.00001	3.08674	0.00265	5.51404	< 0.00001	2.63101	0.01065
Tha_L_8_1	0.536 ± 0.008	0.517 ± 0.010	0.439 ± 0.025	13.64328	< 0.00001	1.52804	0.12979	4.84585	0.00001	3.45964	0.00097
Tha_L_8_4	0.532 ± 0.006	0.493 ± 0.007	0.444 ± 0.015	26.83394	< 0.00001	4.42615	0.00003	6.93617	< 0.00001	3.43972	0.00103
Tha_L_8_5	0.270 ± 0.007	0.237 ± 0.009	0.169 ± 0.020	19.94158	< 0.00001	3.03931	0.00306	5.99811	< 0.00001	3.51982	0.00080
Tha_L_8_6	0.480 ± 0.007	0.437 ± 0.087	0.354 ± 0.021	28.71589	< 0.00001	3.82990	0.00023	7.04932	< 0.00001	4.19047	0.00009
Tha_L_8_7	0.350 ± 0.007	0.312 ± 0.008	0.249 ± 0.021	21.80872	< 0.00001	3.75107	0.00030	6.01745	< 0.00001	3.44748	0.00101
Tha_R_8_1	0.530 ± 0.008	0.507 ± 0.009	0.431 ± 0.022	16.20561	< 0.00001	1.88642	0.06226	5.16813	< 0.00001	3.86826	0.00026
Tha_R_8_5	0.522 ± 0.009	0.488 ± 0.008	0.406 ± 0.022	22.19098	< 0.00001	2.83182	0.00564	6.03756	< 0.00001	4.20599	0.00008
Tha_R_8_6	0.674 ± 0.009	0.632 ± 0.010	0.545 ± 0.022	24.44046	< 0.00001	3.18012	0.00198	6.47704	< 0.00001	4.19148	0.00009
Tha_R_8_8	0.304 ± 0.008	0.281 ± 0.009	0.199 ± 0.025	16.27412	< 0.00001	1.93379	0.05608	5.10105	< 0.00001	3.77282	0.00036
OrG_L_6_5	0.242 ± 0.005	0.214 ± 0.006	0.200 ± 0.007	13.00010	0.00001	3.66359	0.00041	4.63139	0.00001	1.53404	0.12995
Tha_L_8_8	0.088 ± 0.002	0.084 ± 0.003	0.064 ± 0.006	12.24964	0.00001	1.22093	0.22510	4.61960	0.00002	3.48280	0.00090
Tha_R_8_7	0.192 ± 0.005	0.159 ± 0.006	0.146 ± 0.010	13.35539	0.00001	4.12803	0.00008	4.39076	0.00004	1.22662	0.22446
IFG_L_6_2	0.182 ± 0.007	0.158 ± 0.007	0.127 ± 0.010	11.69754	0.00002	2.54754	0.01244	4.68767	0.00001	2.61224	0.01120
Tha_L_8_3	0.045 ± 0.004	0.031 ± 0.004	0.012 ± 0.007	12.20982	0.00002	2.74114	0.00730	4.55103	0.00002	2.62211	0.01091
Tha_R_8_3	0.116 ± 0.004	0.114 ± 0.006	0.071 ± 0.012	11.43398	0.00003	0.22344	0.82367	4.41538	0.00003	3.71550	0.00043
MFG_L_7_3	0.159 ± 0.005	0.141 ± 0.005	0.121 ± 0.007	10.93710	0.00004	2.60169	0.01074	4.43549	0.00003	2.43256	0.01780
INS_L_6_3	0.478 ± 0.007	0.448 ± 0.007	0.420 ± 0.011	10.78722	0.00005	2.86461	0.00513	4.28521	0.00005	2.11393	0.03842
INS_R_6_1	0.189 ± 0.008	0.150 ± 0.006	0.138 ± 0.012	10.50779	0.00006	3.76759	0.00028	3.55751	0.00065	0.91843	0.36184
INS_R_6_3	0.580 ± 0.007	0.550 ± 0.009	0.515 ± 0.013	10.65321	0.00006	2.57219	0.01164	4.46456	0.00003	2.31346	0.02392
MFG_R_7_3	0.111 ± 0.005	0.086 ± 0.005	0.074 ± 0.008	10.63195	0.00006	3.45689	0.00082	3.81831	0.00027	1.35187	0.18118
MFG_R_7_7	0.049 ± 0.006	0.017 ± 0.006	0.011 ± 0.007	10.24133	0.00008	3.66523	0.00041	3.69699	0.00041	0.58544	0.56031
pSTS_L_2_2	0.387 ± 0.007	0.354 ± 0.009	0.332 ± 0.012	9.87506	0.00011	3.14000	0.00225	4.28471	0.00005	1.40169	0.16584
IFG_R_6_5	0.256 ± 0.006	0.241 ± 0.006	0.212 ± 0.008	9.73637	0.00012	1.85850	0.06616	4.28387	0.00005	2.87134	0.00554
IPL_L_6_5	0.156 ± 0.005	0.134 ± 0.007	0.113 ± 0.009	9.50992	0.00015	2.61309	0.01042	4.42417	0.00003	1.87943	0.06474

OrG_L_6_6	0.125 ± 0.005	0.100 ± 0.006	0.080 ± 0.009	9.37504	0.00017	2.65731	0.00923	4.12041	0.00010	1.92711	0.05841

GMVs in the three groups are shown as the mean ± SD (*P* <0.05, Bonferroni corrected). *F*, *F* value of ANOVA; *t*, *t* score of the *post hoc* analysis; GMV, grey matter volume.

BG, basal ganglia; IFG, inferior frontal gyrus; INS, insular gyrus; IPL, inferior parietal lobule; MFG, middle frontal gyrus; OrG, orbital gyrus; PrG, precentral gyrus; pSTS, posterior superior temporal sulcus; Tha, thalamus; L, left; R, right. Detailed information on the brain regions is available at http://atlas.brainnetome.org/.

Table S4. The regions in which significant correlations between mean cortical thickness and cognitive performance were identified in the WMH-MCI patients and the corresponding mean regional cortical thickness in the three groups.

Brain Area	correlation analysis between cortical thickness and processing speed		correlation analysis between cortical thickness and executive function		Mean cortical thickness		
	r	*P*	r	*P*	HC	WMH-nCI	WMH-MCI
OrG_L_6_1	0.694	< 0.001*	0.527	0.010*	3.153 ± 0.013	3.100 ± 0.020	3.016 ± 0.029
STG_R_6_3	0.628	0.001*	0.629	0.001*	3.436 ± 0.020	3.349 ± 0.026	3.269 ± 0.035
PrG_L_6_5	0.592	0.003*	0.560	0.005*	3.351 ± 0.015	3.298 ± 0.019	3.208 ± 0.023
IFG_R_6_5	0.550	0.007*	0.586	0.003*	3.672 ± 0.015	3.584 ± 0.017	3.465 ± 0.032
MFG_R_7_3	0.543	0.007*	0.493	0.017*	3.103 ± 0.012	3.077 ± 0.018	2.935 ± 0.029
STG_L_6_3	0.540	0.008*	0.430	0.040*	3.227 ± 0.020	3.121 ± 0.028	3.063 ± 0.030
MFG_R_7_4	0.532	0.009*	0.602	0.002*	3.047 ± 0.013	3.026 ± 0.018	2.910 ± 0.028
IFG_R_6_2	0.531	0.009*	0.462	0.026*	3.466 ± 0.017	3.436 ± 0.020	3.326 ± 0.028
PoG_L_4_2	0.529	0.009*	0.423	0.044*	3.084 ± 0.016	3.008 ± 0.019	2.931 ± 0.035
FuG_L_3_2	0.509	0.013*	0.471	0.023*	2.925 ± 0.020	2.769 ± 0.024	2.769 ± 0.034
IPL_L_6_5	0.498	0.016*	0.318	0.140	2.912 ± 0.016	2.869 ± 0.018	2.766 ± 0.029
MFG_L_7_4	0.485	0.019*	0.369	0.083*	1.980 ± 0.012	2.939 ± 0.016	2.841 ± 0.030
MFG_L_7_3	0.485	0.019*	0.480	0.020*	3.001 ± 0.014	2.982 ± 0.021	2.832 ± 0.034
IPL_R_6_6	0.479	0.021*	0.427	0.042*	3.110 ± 0.013	3.053 ± 0.018	2.980 ± 0.025
IFG_L_6_2	0.472	0.023*	0.373	0.080	3.196 ± 0.015	3.141 ± 0.021	3.008 ± 0.035
SFG_L_7_7	0.458	0.028*	0.422	0.045*	3.358 ± 0.013	3.313 ± 0.020	3.202 ± 0.028
STG_R_6_6	0.455	0.029*	0.524	0.010*	3.460 ± 0.019	3.375 ± 0.023	3.278 ± 0.030
PoG_R_4_2	0.441	0.035*	0.466	0.025*	2.969 ± 0.015	2.903 ± 0.017	2.835 ± 0.030
IPL_L_6_6	0.438	0.037*	0.360	0.091	3.109 ± 0.013	3.044 ± 0.020	2.957 ± 0.030
INS_L_6_6	0.434	0.039*	0.486	0.019*	3.895 ± 0.021	3.740 ± 0.036	3.707 ± 0.046
IFG_L_6_4	0.431	0.040*	0.468	0.024*	2.992 ± 0.015	2.935 ± 0.020	2.858 ± 0.023
INS_L_6_5	0.430	0.040*	0.548	0.007*	3.452 ± 0.030	3.280 ± 0.040	3.208 ± 0.049
MFG_L_7_2	0.416	0.048*	0.477	0.002*	3.127 ± 0.014	3.108 ± 0.020	2.979 ± 0.034
OrG_L_6_6	0.412	0.005*	0.590	0.003*	3.259 ± 0.017	3.201 ± 0.018	3.121 ± 0.028
SFG_R_7_6	0.409	0.053	0.574	0.004*	3.497 ± 0.015	3.485 ± 0.019	3.364 ± 0.032
SFG_R_7_7	0.373	0.080	0.476	0.022*	3.344 ± 0.013	3.306 ± 0.021	3.211 ± 0.032

IFG_L_6_5	0.360	0.092	0.518	0.011*	3.582 ± 0.016	3.504 ± 0.020	3.373 ± 0.042
SFG_L_7_6	0.343	0.109	0.627	0.001*	3.451 ± 0.013	3.414 ± 0.020	3.326 ± 0.030
IFG_R_6_1	0.264	0.223	0.427	0.042*	3.147 ± 0.020	3.080 ± 0.024	2.963 ± 0.043
INS_R_6_1	0.181	0.408	0.445	0.033*	3.595 ± 0.026	3.436 ± 0.033	3.299 ± 0.075
CG_L_7_3	0.144	0.511	0.420	0.046*	3.775 ± 0.024	3.715 ± 0.030	3.566 ± 0.044

*significant associations (P <0.05) between regional mean cortical thickness and cognition (processing speed or executive function).

CG, cingulate gyrus; FuG, fusiform gyrus; IFG, inferior frontal gyrus; INS, insular gyrus; IPL, inferior parietal lobule; MFG, middle frontal gyrus; OrG, orbital gyrus; PrG, precentral gyrus; PoG, postcentral gyrus; SFG, superior frontal gyrus; STG, superior temporal gyrus; L, left; R, right. Detailed information on the brain regions is available at http://atlas.brainnetome.org.

Table S5. Regions in which significant correlations between mean grey matter volume and cognitive performance were identified in WMH-MCI patients and the corresponding mean regional grey matter volume in the three groups.

Brain Area	Correlation analysis between GMV and processing speed		Correlation analysis between GMV and executive function		Mean cortical thickness		
	r	*P*	r	*P*	HC	WMH-nCI	WMH-MCI
Tha_L_8_6	0.482	0.020*	0.491	0.017*	0.480 ± 0.007	0.437 ± 0.087	0.354 ± 0.021
IFG_L_6_2	0.442	0.034*	0.249	0.252	0.182 ± 0.007	0.158 ± 0.007	0.127 ± 0.010
Tha_R_8_5	0.359	0.092	0.508	0.013*	0.522 ± 0.009	0.488 ± 0.008	0.406 ± 0.022
Tha_R_8_3	0.314	0.144	0.528	0.010*	0.116 ± 0.004	0.114 ± 0.006	0.071 ± 0.012
Tha_R_8_6	0.278	0.200	0.447	0.033*	0.674 ± 0.009	0.632 ± 0.010	0.545 ± 0.022

*significant associations (P <0.05) between regional mean GMV and cognition (processing speed or executive function). GMV, grey matter volume.

IFG, inferior frontal gyrus; Tha, thalamus; L, left; R, right. Detailed information on the brain regions is available at http://atlas.brainnetome.org/.

Table S6. Mediation effect of cortical thickness on the relationship between WMH and cognition.

ROI of mediators	Path a		Path b		Direct effect (path c')		Mediation effect (path a*b)	
	β	*P*	β	*P*	β	*P*	β	95%CI
**Mediation effect of cortical thickness on the association between WMH and processing speed**								
**Total effect: β = –0.742 p <0.001**								
SFG_L_7_7	-0.083	0.009	2.082	0.014	-0.569	0.008	-0.173	(-0.395, -0.034)
MFG_L_7_3	-0.126	< 0.001	2.787	< 0.001	-0.390	0.063	-0.352	(-0.664, -0.121)
MFG_R_7_3	-0.085	0.006	3.116	< 0.001	-0.476	0.017	-0.266	(-0.524, -0.082)
MFG_R_7_4	-0.087	0.004	3.643	< 0.001	-0.426	0.029	-0.316	(-0.596, -0.095)
IFG_L_6_2	-0.088	0.015	2.443	< 0.001	-0.527	0.009	-0.215	(-0.527, -0.031)
IFG_L_6_5	-0.108	0.004	1.433	0.050	-0.587	0.008	-0.155	(-0.430, -0.023)
IFG_R_6_5	-0.111	< 0.001	2.860	0.002	-0.423	0.050	-0.319	(-0.626, -0.097)
MFG_L_7_2	-0.064	0.053	2.839	<0.001	-0.560	0.004	-0.182	(-0.396, -0.003)
OrG_L_6_1	-0.087	0.029	2.448	0.006	-0.529	0.013	-0.213	(-0.495, -0.039)
PrG_L_6_5	-0.089	0.002	2.484	0.009	-0.521	0.016	-0.221	(-0.561, -0.040)
IPL_R_6_6	-0.064	0.019	2.447	0.012	-0.585	0.006	-0.157	(-0.381, -0.016)
PoG_L_4_2	-0.078	0.015	2.056	0.014	-0.582	0.006	-0.160	(-0.369, -0.010)
IFG_R_6_1	-0.087	0.029	1.664	0.012	-0.597	0.005	-0.145	(-0.408, -0.003)
IFG_L_6_4	-0.077	0.005	2.386	0.016	-0.559	0.010	-0.183	(-0.378, -0.036)
PoG_R_4_2	-0.071	0.012	2.154	0.025	-0.589	0.006	-0.153	(-0.356, -0.014)
SFG_R_7_6	-0.067	0.036	1.977	0.017	-0.609	0.004	-0.133	(-0.346, -0.003)
STG_R_6_3	-0.096	0.010	1.357	0.063	-0.612	0.005	-0.130	(-0.309, -0.007)
MFG_L_7_7	-0.085	0.007	1.770	0.041	-0.592	0.007	-0.150	(-0.471, -0.001)
**Mediation effect of cortical thickness on the association between WMH and executive function**								
**Total effect: β = –0.509 *P* = 0.003**								
SFG_L_7_7	-0.083	0.009	1.930	0.005	-0.349	0.038	-0.161	(-0.331, -0.036)
MFG_L_7_3	-0.126	< 0.001	2.267	< 0.001	-0.223	0.185	-0.287	(-0.506, -0.116)
MFG_R_7_3	-0.085	0.006	2.763	< 0.001	-0.274	0.079	-0.235	(-0.445, -0.084)
MFG_R_7_4	-0.087	0.004	3.017	< 0.001	-0.248	0.109	-0.261	(-0.487, -0.077)
IFG_L_6_2	-0.088	0.015	1.768	0.003	-0.354	0.033	-0.156	(-0.367, -0.027)
IFG_L_6_5	-0.108	0.004	1.569	0.007	-0.340	0.047	-0.169	(-0.385, -0.015)
IFG_R_6_5	-0.111	< 0.001	1.985	0.007	-0.288	0.105	-0.221	(-0.486, -0.034)

STG_R_6_6	-0.077	0.024	1.126	0.078	-0.423	0.015	-0.087	(-0.234, -0.002)
IPL_L_6_6	-0.080	0.010	1.300	0.069	-0.406	0.021	-0.104	(-0.271, -0.003)
INS_L_6_1	-0.125	0.017	0.744	0.075	-0.416	0.017	-0.093	(-0.242, -0.004)
MFG_L_7_2	-0.064	0.053	2.336	< 0.001	-0.360	0.022	-0.149	(-0.295, -0.007)
OrG_L_6_1	-0.087	0.004	1.777	0.015	-0.355	0.041	-0.155	(-0.350, -0.023)
PrG_L_6_5	-0.089	0.002	2.231	0.004	-0.311	0.069	-0.198	(-0.445, -0.042)
IPL_R_6_6	-0.064	0.019	1.925	0.015	-0.386	0.023	-0.123	(-0.294, -0.012)
PoG_L_4_2	-0.078	0.015	1.651	0.015	-0.381	0.025	-0.128	(-0.285, -0.011)
IFG_L_6_4	-0.077	0.005	1.848	0.022	-0.368	0.034	-0.141	(-0.297, -0.030)
PoG_R_4_2	-0.071	0.012	2.024	0.008	-0.366	0.031	-0.143	(-0.309, -0.014)
SFG_R_7_6	-0.067	0.036	2.244	<0.001	-0.358	0.025	-0.151	(-0.329, -0.021)
SFG_R_7_7	-0.076	0.023	1.710	0.008	-0.380	0.024	-0.129	(-0.298, -0.020)
MFG_L_7_7	-0.085	0.007	1.706	0.014	-0.365	0.034	-0.145	(-0.381, -0.018)

β, standardized regression coefficient; CI, confidence interval; X, predictor variable; Y, outcome variable; M, mediator; TIV, total intracranial volume. IFG, inferior frontal gyrus; INS, insular gyrus; IPL, inferior parietal lobule; MFG, middle frontal gyrus; OrG, orbital gyrus; PoG, postcentral gyrus; PrG, precentral gyrus; SFG, superior frontal gyrus; STG, superior temporal gyrus; L, left; R, right.

The table shows the areas in which cortical thickness was confirmed to mediate the relationship between WMH and cognition. Mediation analysis was used to assess the potential indirect relationship between log-transformed WMH volume (X) and cognitive performance (Y) *via* mean cortical thickness in regions identified by between-group analyses (M). In each model, age, sex, years of education, and TIV were entered as covariates. Paths a and b indicate the association between WMH and cortical thickness, and the associations between cortical thickness and cognition (when both WMH and cortical thickness were entered into the model as predictive variables), respectively. Path c represents the total effect of WMH on cognition, and path c' shows the direct effect of WMH on cognition after controlling for cortical thickness as a mediating factor. The

mediating role of cortical thickness on the association between WMH and cognition is defined by the 95% bootstrap CI for 5,000 bootstrapping iterations. Significant mediation effects were defined by a 95% CI entirely above or below 0.

Table S7. Mediation effect of grey matter volume on the relationship between WMH and cognition.

ROI of mediators	Path a		Path b		Direct effect (path c')		Mediation effect (path a*b)	
	β	*P*	β	*P*	β	*P*	β	95%CI
**Mediation effect of GMV on the association between WMH and processing speed**<br>**Total effect: β = –0.742 *P* <0.001**								
IFG_L_6_2	-0.028	0.009	7.419	0.003	-0.537	0.010	-0.205	(-0.517, -0.005)
IFG_R_6_5	-0.026	0.005	8.175	0.006	-0.533	0.012	-0.209	(-0.396, -0.058)
INS_R_6_3	-0.031	0.022	5.307	0.007	-0.577	0.006	-0.165	(-0.370, -0.023)
OrG_L_6_5	-0.023	0.005	7.000	0.010	-0.509	0.003	-0.158	(-0.379, -0.017)
Tha_L_8_1	-0.045	0.029	4.460	< 0.001	-0.539	0.007	-0.203	(-0.392, -0.044)
Tha_L_8_4	-0.048	< 0.001	8.573	< 0.001	-0.332	0.109	-0.410	(-0.738, -0.171)
Tha_L_8_5	-0.042	0.019	5.315	< 0.001	-0.521	0.009	-0.221	(-0.463, -0.041)
Tha_L_8_6	-0.060	0.001	6.109	< 0.001	-0.374	0.056	-0.368	(-0.669, -0.124)
Tha_L_8_7	-0.049	0.003	5.401	< 0.001	-0.479	0.020	-0.263	(-0.501, -0.077)
Tha_R_8_1	-0.051	0.004	4.256	0.005	-0.524	0.014	-0.218	(-0.440, -0.054)
Tha_R_8_5	-0.059	< 0.001	6.055	< 0.001	-0.382	0.054	-0.360	(-0.665, -0.128)
Tha_R_8_6	-0.072	< 0.001	5.519	< 0.001	-0.345	0.097	-0.397	(-0.695, -0.160)
**Mediation effect of GMV on the association between WMH and executive function**<br>**Total effect: β = –0.509 *P* = 0.003**								
IFG_R_6_5	-0.026	0.005	5.348	0.029	-0.373	0.033	-0.137	(-0.316, -0.007)
INS_R_6_3	-0.031	0.022	4.313	0.006	-0.375	0.025	-0.134	(-0.291, -0.018)
OrG_L_6_5	-0.023	0.005	6.996	0.010	-0.351	0.041	-0.158	(-0.369, -0.017)
Tha_L_8_1	-0.045	0.029	3.358	< 0.001	-0.357	0.027	-0.153	(-0.327, -0.026)
Tha_L_8_4	-0.048	< 0.001	6.355	< 0.001	-0.205	0.230	-0.304	(-0.582, -0.115)
Tha_L_8_5	-0.042	0.019	3.821	0.002	-0.350	0.032	-0.159	(-0.365, -0.023)
Tha_L_8_6	-0.060	0.001	4.328	< 0.001	-0.249	0.130	-0.260	(-0.522, -0.077)

Tha_L_8_7	-0.049	0.003	4.049	0.002	-0.312	0.063	-0.197	(-0.390, -0.051)
Tha_R_8_1	-0.051	0.004	3.666	0.003	-0.321	0.056	-0.188	(-0.377, -0.052)
Tha_R_8_5	-0.059	< 0.001	4.923	< 0.001	-0.217	0.172	-0.292	(-0.561, -0.095)
Tha_R_8_6	-0.072	< 0.001	4.457	< 0.001	-0.189	0.258	-0.320	(-0.584, -0.117)

The table shows the areas in which grey matter volume (GMV) was confirmed to mediate the relationship between WMH and cognition. Mediation analysis was used to assess the potential indirect relationship between log-transformed WMH volume (X) and cognitive performance (Y) *via* mean GMV in regions identified by between-group analyses (M). In each model, age, sex, years of education, and TIV were entered as covariates. Paths a and b indicate the association between WMH and GMV, and the associations between GMV and cognition (when both WMH and GMV were entered into the model as predictive variables), respectively. Path c represents the total effect of WMH on cognition, and path c' shows the direct effect of WMH on cognition after controlling for GMV as a mediating factor. The mediating role of the GMV on the association between WMH and cognition is defined by the 95% bootstrap CI for 5,000 bootstrapping iterations. Significant mediation effects were defined by a 95% CI entirely above or below 0.

β, Standardized regression coefficient; CI, confidence interval; X, predictor variable; Y, outcome variable; M, mediator; GMV, grey matter volume; TIV, total intracranial volume; IFG, inferior frontal gyrus; INS, insular gyrus; OrG, orbital gyrus; Tha, thalamus; L, left; R, right.

Table S8. Differences in cortical thickness among the WMH-MCI, WMH-NCI, and HC groups in the in-house dataset at the voxel level.

Region	Cluster size (voxels)	MNI space			Peak *F*-values	Mean cortical thickness		
		X	Y	Z		HC	WMH-nCI	WMH-MCI
Insula_R	287	37	-22	-1	16.41	2.67 ± 0.11	2.56 ± 0.13[a]	2.47 ± 0.20[b,c]
Rolandic_oper_L/Heschl_L	197	-43	-22	16	15.79	2.93 ± 0.11	2.97 ± 0.16[a]	2.71 ± 0.21[b]
		-59	-11	10	16.95			
Frontal_sup_orb_R/Frontal_mid_R	131	20	60	-4	14.42	1.97 ± 0.18	1.83 ± 0.18[a]	1.73 ± 0.20[b,c]
		39	50	15	13.70			
Frontal_inf_Tri_L	125	-50	35	-1	19.02	3.09 ± 0.20	2.88 ± 0.17[a]	2.84 ± 0.25[b]
Insula_R	96	36	23	9	16.50	2.50 ± 0.10	2.46 ± 0.12[a]	2.32 ± 0.18[b,c]
Postcentral_L	80	-41	-22	37	15.39	2.96 ± 0.14	2.84 ± 0.14[a]	2.73 ± 0.23[b,c]

All regions survived clusterwise-correction in ANOVA with age, sex, and years of education as covariates (P <0.01, FDR corrected). Post-hoc analyses were performed in mean grey matter density of the clusters identified between each two group with age, sex, and years of education as covariates (P <0.05, FDR corrected).

[a] Significant difference between the WMH-nCI and HC groups.

[b] Significant difference between the WMH-MCI and HC groups.

[c] Significant difference between the WMH-MCI and WMH-nCI groups.

Table S9. Differences in grey matter volume among the WMH-MCI, WMH-NCI, and HC groups in the in-house dataset at the voxel level.

Region	Cluster size (voxels)	MNI space			Peak *F*-values	Mean grey matter volume		
		X	Y	Z		HC	WMH-nCI	WMH-MCI
Left/Right THA	5529	-22.5	-31.5	2.5	25.63	0.60 ± 0.07	0.55 ± 0.05[a]	0.46 ± 0.12[b,c]
		5.5	-8.5	12.5	24.95			
Right CAU	3013	18.5	24.5	10.5	68.26	0.39 ± 0.06	0.50 ± 0.09[a]	0.51 ± 0.10[b]
Left CAU	1255	-17.5	-26.5	-1.5	76.00	0.30 ± 0.05	0.40 ± 0.07[a]	0.43 ± 0.09[b]
Right INS/IFG	353	32.5	26.5	0.5	20.51	0.56 ± 0.07	0.52 ± 0.07[a]	0.46 ± 0.07[b,c]
Left INS	186	-33.5	18.5	4.5	16.45	0.58 ± 0.07	0.54 ± 0.06[a]	0.48 ± 0.07[b,c]
Left PRG	149	-55.5	-6.5	36.5	15.87	0.38 ± 0.06	0.34 ± 0.07[a]	0.29 ± 0.06[b,c]
Left PCUN	78	-16.5	-44.5	31.5	21.53	0.10 ± 0.03	0.16 ± 0.12[a]	0.24 ± 0.16[b,c]

All regions survived cluster wise-correction in ANOVA with age, sex, years of education, and TIV as covariates (P <0.01, FDR corrected). *Post-hoc* analysis was performed in mean grey matter density of the clusters identified between each of two groups with age, sex, years of education, and TIV as covariates (P <0.05, FDR corrected). TIV, total intracranial volume. CAU, caudate; IFG, inferior frontal gyrus; INS, insular gyrus; PRG, precentral gyrus; PCUN, precuneus; TIV, total intracranial volume.

[a] Significant difference between the WMH-nCI and HC groups.

[b] Significant difference between the WMH-MCI and HC groups.

[c] Significant difference between the WMH-MCI and WMH-nCI groups.

Table S10. Epidemiologic data of participants in the replication dataset from ADNI.

	HC (n = 69)	WMH-nCI (n = 23)	WMH-MCI (n = 62)	Overall P Value
Age (years)	72.89 ± 6.18	76.91 ± 6.65[d]	76.19 ± 6.60[e]	0.004[a]
Gender (M/F)	36/33	14/9	31/31	0.669[b]
Education (years)	16.67 ± 2.68	16.57 ± 2.59	15.52 ± 2.90	0.064[a]
TIV (cm^3)	1360.79 ± 139.00	1413.74 ± 142.23	1378.06 ± 138.71	0.050[c]
Fazekas WMH score	1.62 ± 0.67	4.17 ± 1.15[d]	4.29 ± 1.06[e]	< 0.001[a]
Total WMH volume (cm^3)	2.21 ± 1.53	16.34 ± 16.79[d]	17.46 ± 13.92[e]	< 0.001[a]
MMSE	29.19 ± 1.13	29.00 ± 1.45	27.60 ± 1.78[e,f]	< 0.001[a]
Executive function [g]	1.13 ± 0.91	0.59 ± 0.84	-0.04 ± 0.72[e,f]	< 0.001[c]
Memory [g]	1.17 ± 0.63	0.91 ± 0.60[d]	0.26 ± 0.69[e,f]	< 0.001[c]

[a] Kruskal-Wallis test.

[b] χ^2 test.

[c] One-way analysis of variance.

[d] Significant difference between the WMH-nCI and HC groups.

[e] Significant difference between the WMH-MCI and HC groups.

[f] Significant difference between the WMH-MCI and WMH-nCI groups.

[g] Z-scores.

TIV, total intracranial volume; MMSE, mini-mental state examination.

Table S11. Regional differences in cortical thickness among the WMH-MCI, WMH-nCI, and HC groups identified by the replication dataset from ADNI.

Brain Area	Mean cortical thickness			ANOVA		HC vs WMH-nCI		HC vs WMH-MCI		WMH-nCI vs WMH-	
	**HC**	**WMH-nCI**	**WMH-MCI**	***F***	***P***	***t***	***P***	***t***	***P***	***t***	***P***
SFG L 7 6	3.066 ± 0.017	2.971 ± 0.028	2.890 ± 0.020	23.5558	< 0.00001	2.79229	0.0063	6.7790	< 0.00001	2.21446	0.02954
SFG_R_7_6	4.125 ± 0.029	3.943 ± 0.049	3.835 ± 0.032	23.2216	< 0.00001	3.15753	0.0021	6.7338	< 0.00001	1.78079	0.07861
SFG_L_7_7	4.497 ± 0.044	4.186 ± 0.064	4.084 ± 0.046	22.7297	< 0.00001	3.64022	0.0004	6.4520	< 0.00001	1.19651	0.23491
SFG_R_7_7	3.386 ± 0.022	3.250 ± 0.053	3.156 ± 0.024	22.6801	< 0.00001	2.78839	0.0064	7.1295	< 0.00001	1.87209	0.06472
MFG_L_7_1	3.349 ± 0.019	3.220 ± 0.033	3.16 ± 0.021	22.4410	< 0.00001	3.42661	0.0009	6.5881	< 0.00001	1.39204	0.16763
MFG_L_7_3	4.140 ± 0.047	3.881 ± 0.071	3.728 ± 0.044	21.0393	< 0.00001	2.85820	0.0052	6.3743	< 0.00001	1.80409	0.07485
MFG_R_7_3	3.051 ± 0.021	2.968 ± 0.037	2.854 ± 0.023	19.9196	< 0.00001	1.94320	0.0551	6.3065	< 0.00001	2.59848	0.01108
MFG_L_7_4	3.034 ± 0.016	2.937 ± 0.042	2.870 ± 0.019	19.6954	< 0.00001	2.68039	0.0087	6.6995	< 0.00001	1.67038	0.09861
MFG_R_7_4	3.868 ± 0.032	3.723 ± 0.060	3.516 ± 0.049	19.5023	< 0.00001	2.24285	0.0273	6.1284	< 0.00001	2.32787	0.02235
IFG_R_6_2	3.710 ± 0.020	3.615 ± 0.038	3.517 ± 0.023	19.4460	< 0.00001	2.29078	0.0243	6.2730	< 0.00001	2.18917	0.03139
IFG_R_6_3	3.344 ± 0.021	3.259 ± 0.036	3.144 ± 0.026	18.6696	< 0.00001	1.99216	0.0493	6.0350	< 0.00001	2.42514	0.01747
IFG_R_6_4	3.357 ± 0.022	3.300 ± 0.042	3.160 ± 0.025	18.0428	< 0.00001	1.28490	0.2021	6.0250	< 0.00001	2.89972	0.00478
IFG_L_6_5	2.980 ± 0.022	2.859 ± 0.036	2.796 ± 0.021	18.0028	< 0.00001	2.74995	0.0072	5.9263	< 0.00001	1.51462	0.13367
IFG_R_6_6	3.286 ± 0.018	3.200 ± 0.035	3.128 ± 0.018	17.6943	< 0.00001	2.28523	0.0246	6.0676	< 0.00001	1.93702	0.05615
OrG_L_6_1	3.461 ± 0.027	3.251 ± 0.069	3.221 ± 0.029	17.5209	< 0.00001	3.44942	0.0008	6.11002	< 0.00001	0.49068	0.62494
OrG_L_6_3	2.943 ± 0.019	2.837 ± 0.045	2.765 ± 0.022	17.2256	< 0.00001	2.51165	0.0138	6.1473	< 0.00001	1.58562	0.11663
PrG_L_6_5	2.865 ± 0.019	2.708 ± 0.038	2.708 ± 0.021	17.0946	< 0.00001	4.00246	0.0001	5.5002	< 0.00001	-0.01023	0.99186
PrG_R_6_5	4.108 ± 0.068	3.961 ± 0.087	3.576 ± 0.066	17.0878	< 0.00001	1.14790	0.2540	5.6048	< 0.00001	3.19515	0.00198
STG_L_6_1	2.750 ± 0.023	2.623 ± 0.041	2.558 ± 0.023	17.0839	< 0.00001	2.71918	0.0078	5.8296	< 0.00001	1.43046	0.15634
STG_R_6_1	3.845 ± 0.061	3.538 ± 0.091	3.277 ± 0.082	17.0641	< 0.00001	2.61491	0.0104	5.6482	< 0.00001	1.79254	0.07669
STG_L_6_3	3.154 ± 0.019	3.055 ± 0.037	2.978 ± 0.024	16.99311	< 0.00001	2.54231	0.0127	5.8488	< 0.00001	1.69828	0.09320
STG_R_6_3	3.184 ± 0.020	3.060 ± 0.040	2.999 ± 0.025	16.9089	< 0.00001	2.94602	0.0041	5.7975	< 0.00001	1.27989	0.20415
STG_L_6_5	4.592 ± 0.042	4.455 ± 0.070	4.234 ± 0.046	16.8369	< 0.00001	1.63341	0.1058	5.7455	< 0.00001	2.55177	0.01255
STG_R_6_5	3.110 ± 0.019	2.997 ± 0.040	2.943 ± 0.022	16.5989	< 0.00001	2.85762	0.0053	5.8390	< 0.00001	1.24934	0.21505
STG_R_6_6	3.011 ± 0.023	2.864 ± 0.042	2.818 ± 0.025	16.5032	< 0.00001	3.12563	0.0023	5.6359	< 0.00001	0.94157	0.34915
MTG_L_4_2	3.077 ± 0.019	3.027 ± 0.029	2.932 ± 0.017	16.3960	< 0.00001	1.35082	0.1801	5.6672	< 0.00001	2.89733	0.00481
MTG_R_4_4	3.105 ± 0.017	2.936 ± 0.039	2.955 ± 0.024	15.9275	< 0.00001	4.52443	0.0000	5.1293	< 0.00001	-0.42085	0.67495

FuG_R_3_2	2.943 ± 0.018	2.846 ± 0.033	2.797 ± 0.019	15.8459	< 0.00001	2.69821	0.0083	5.6156	< 0.00001	1.31721	0.19139
PhG_L_6_4	3.903 ± 0.034	3.750 ± 0.051	3.602 ± 0.044	15.7969	< 0.00001	2.33163	0.0219	5.4493	< 0.00001	1.86600	0.06557
PhG_L_6_5	2.874 ± 0.026	2.815 ± 0.044	2.678 ± 0.023	15.5307	< 0.00001	1.12771	0.2624	5.5566	< 0.00001	2.96313	0.00397
pSTS_R_2_1	2.432 ± 0.023	2.329 ± 0.046	2.238 ± 0.025	15.4684	< 0.00001	2.12399	0.0364	5.6704	< 0.00001	1.83050	0.07076
IPL_L_6_6	3.256 ± 0.018	3.133 ± 0.037	3.114 ± 0.019	14.6248	< 0.00001	3.19606	0.0019	5.3150	< 0.00001	0.49069	0.62494
INS_L_6_2	4.281 ± 0.028	4.159 ± 0.049	4.059 ± 0.031	14.55211	< 0.00001	2.18050	0.0318	5.3826	< 0.00001	1.71467	0.09014
INS_R_6_4	2.805 ± 0.017	2.719 ± 0.034	2.665 ± 0.020	14.4132	< 0.00001	2.40644	0.0181	5.4233	< 0.00001	1.43352	0.15546
INS_L_6_6	3.455 ± 0.018	3.376 ± 0.038	3.303 ± 0.022	13.4855	<0.00001	2.03067	0.0452	5.2773	< 0.00001	1.68756	0.09525
MFG_R_7_2	3.636 ± 0.041	3.504 ± 0.069	3.322 ± 0.046	13.2462	0.00001	1.62318	0.1080	5.1016	< 0.00001	2.09036	0.03965
IFG_L_6_2	3.055 ± 0.022	2.968 ± 0.038	2.889 ± 0.023	13.2264	0.00001	1.95546	0.0536	5.1308	< 0.00001	1.76046	0.08201
PrG_L_6_1	2.788 ± 0.023	2.617 ± 0.033	2.628 ± 0.027	13.2213	0.00001	3.90343	0.0001	4.5506	0.00001	-0.20351	0.83923
MTG_L_4_4	3.502 ± 0.033	3.388 ± 0.066	3.241 ± 0.038	13.1613	0.00001	1.65548	0.1013	5.2199	< 0.00001	1.98218	0.05077
PhG_L_6_1	2.810 ± 0.017	2.686 ± 0.030	2.693 ± 0.018	12.9297	0.00001	3.57701	0.0005	4.63113	0.00001	-0.20217	0.84028
IPL_R_6_6	2.795 ± 0.022	2.678 ± 0.034	2.647 ± 0.020	12.8662	0.00001	2.72379	0.0077	4.8848	< 0.00001	0.76816	0.44457
INS_R_6_2	3.004 ± 0.018	2.883 ± 0.033	2.877 ± 0.019	12.7319	0.00001	3.27557	0.0015	4.7568	0.00001	0.14891	0.88199
INS_L_6_5	2.571 ± 0.021	2.465 ± 0.033	2.423 ± 0.022	12.3520	0.00001	2.58198	0.01144	4.8202	< 0.00001	1.00151	0.31949
CG_L_7_6	4.230 ± 0.055	4.058 ± 0.077	3.833 ± 0.064	11.99050	0.00001	1.64055	0.1043	4.7501	0.00001	1.95797	0.05359
IFG_R_6_5	3.389 ± 0.020	3.259 ± 0.041	3.252 ± 0.022	11.61057	0.00002	3.09358	0.0026	4.6450	0.00001	0.17661	0.86025
OrG_R_6_3	2.816 ± 0.020	2.681 ± 0.050	2.665 ± 0.025	11.27286	0.00003	2.95961	0.0039	4.7683	< 0.00001	0.31924	0.75034
MFG_R_7_5	3.123 ± 0.022	2.988 ± 0.040	2.982 ± 0.024	10.7152	0.00004	3.05924	0.0029	4.3501	0.00003	0.13191	0.89537
SFG_L_7_3	3.316 ± 0.030	3.187 ± 0.059	3.124 ± 0.026	10.6835	0.00005	2.06647	0.0416	4.7285	0.00001	1.11533	0.26793
MFG_R_7_7	3.121 ± 0.023	3.056 ± 0.037	2.956 ± 0.029	10.6448	0.00005	1.4525	0.1498	4.5130	0.00001	1.88481	0.06296
FuG_L_3_1	2.896 ± 0.021	2.818 ± 0.048	2.744 ± 0.024	10.5813	0.00005	1.70885	0.0909	4.7987	< 0.00001	1.51782	0.13286
OrG_L_6_5	3.137 ± 0.020	3.005 ± 0.044	2.990 ± 0.027	10.4605	0.00006	3.05698	0.0029	4.4181	0.00002	0.28323	0.77770
FuG_L_3_2	2.193 ± 0.017	2.093 ± 0.034	2.085 ± 0.018	10.3195	0.00006	2.80477	0.0061	4.3818	0.00002	0.22191	0.82493
PCun_R_4_3	2.629 ± 0.023	2.534 ± 0.041	2.479 ± 0.024	10.3097	0.00006	2.05696	0.0425	4.5354	0.00001	1.18470	0.23952
MFG_L_7_7	2.982 ± 0.021	2.888 ± 0.042	2.842 ± 0.022	10.2797	0.00007	2.17001	0.0326	4.5873	0.00001	1.02214	0.30969
INS_L_6_1	3.308 ± 0.027	3.174 ± 0.041	3.141 ± 0.029	10.0703	0.00008	2.59561	0.01103	4.2733	0.00004	0.61388	0.54098
INS_R_6_5	2.734 ± 0.035	2.428 ± 0.069	2.574 ± 0.039	10.0543	0.00008	4.2133	0.0000	3.0317	0.00294	-1.90377	0.06041
IFG_L_6_3	3.156 ± 0.022	3.052 ± 0.041	3.012 ± 0.024	9.94867	0.00009	2.30694	0.0233	4.4147	0.00002	0.85566	0.39465
pSTS_L_2_1	2.744 ± 0.021	2.683 ± 0.038	2.607 ± 0.022	9.93978	0.00009	1.42695	0.1570	4.4821	0.00002	1.77260	0.07996
ITG_L_7_3	3.807 ± 0.033	3.791 ± 0.051	3.596 ± 0.040	9.67333	0.00011	0.25139	0.8020	4.1091	0.00007	2.66636	0.00922
IPL_L_6_3	2.682 ± 0.019	2.569 ± 0.037	2.573 ± 0.018	9.53258	0.00013	2.86043	0.0052	4.1206	0.00007	-0.1042	0.91726

CG_L_7_1	3.329 ± 0.025	3.186 ± 0.048	3.181 ± 0.025	9.46900	0.00013	2.76413	0.0069	4.1399	0.00006	0.10354	0.91778
INS_L_6_3	4.095 ± 0.033	3.839 ± 0.071	3.903 ± 0.041	9.36491	0.00015	3.63606	0.0004	3.6800	0.00034	-0.80907	0.42079
MFG_L_7_5	3.218 ± 0.018	3.185 ± 0.032	3.094 ± 0.024	9.12645	0.00018	0.90139	0.3697	4.1570	0.00006	2.06330	0.04221
OrG_R_6_1	2.754 ± 0.019	2.668 ± 0.034	2.628 ± 0.024	9.04909	0.00019	2.23663	0.0277	4.1664	0.00006	0.90463	0.36828
pSTS_L_2_2	2.563 ± 0.019	2.443 ± 0.043	2.445 ± 0.022	9.03117	0.00020	2.93128	0.0042	4.0733	0.00008	-0.04905	0.96100
CG_R_7_1	2.763 ± 0.026	2.611 ± 0.057	2.621 ± 0.023	8.86089	0.00023	2.71058	0.0080	4.0662	0.00008	-0.18755	0.85168

Regional cortical thickness in the three groups are shown as the means ± SD (*P* 0.05, Bonferroni corrected). *F*, *F* value of the ANOVA; *t*, *t* score of the *post hoc* analysis.

CG, cingulate gyrus; FuG, fusiform gyrus; IFG, inferior frontal gyrus; INS, insular gyrus; IPL, Inferior parietal lobule; ITG, inferior temporal gyrus; MFG, middle frontal gyrus; MTG, middle temporal gyrus; OrG, orbital gyrus; Pcun, precuneus; PhG, parahippocampal gyrus; PrG, precentral gyrus; pSTS, posterior superior temporal sulcus; SFG, superior frontal gyrus; STG, superior temporal gyrus; L, left; R, right. Detailed information on the brain regions is available at http://atlas.brainnetome.org.

Table S12. Regional differences in grey matter volume among the WMH-MCI, WMH-nCI, and HC groups identified in the replication dataset from ADNI.

Brain Area	Mean GMV			ANOVA		HC vs WMH-nCI		HC vs WMH-MCI		WMH-nCI vs WMH-	
	HC	WMH-nCI	WMH-MCI	*F*	*P*	*t*	*P*	*t*	*P*	*t*	*P*
Tha_L_8_4	0.869 ± 0.006	0.860 ± 0.011	0.785 ± 0.008	44.72787	< 0.00001	0.79078	0.43115	9.15906	< 0.00001	5.30783	< 0.00001
Tha_R_8_7	0.653 ± 0.005	0.641 ± 0.008	0.582 ± 0.007	42.61833	< 0.00001	1.28924	0.20062	8.77688	< 0.00001	4.92563	<0.00001
Tha_L_8_7	0.848 ± 0.007	0.816 ± 0.014	0.758 ± 0.010	29.62617	< 0.00001	2.22736	0.02842	7.64977	< 0.00001	3.20267	0.00193
Hipp_L_2_2	0.888 ± 0.006	0.873 ± 0.11	0.818 ± 0.008	26.24439	< 0.00001	1.30094	0.19660	6.99991	< 0.00001	3.59020	0.00056
Tha_R_8_4	0.877 ± 0.008	0.881 ± 0.012	0.803 ± 0.008	26.00143	< 0.00001	-0.23961	0.81117	6.62646	< 0.00001	5.10160	< 0.00001
Hipp_R_2_2	0.958 ± 0.007	0.934 ± 0.012	0.880 ± 0.009	25.70706	< 0.00001	1.78536	0.07757	7.05694	< 0.00001	3.25009	0.00167
Hipp_R_2_1	0.736 ± 0.007	0.726 ± 0.012	0.660 ± 0.010	24.56423	< 0.00001	0.79714	0.42747	6.68750	< 0.00001	3.74991	0.00033
Tha_R_8_1	0.880 ± 0.008	0.870 ± 0.015	0.798 ± 0.010	24.19215	< 0.00001	0.64162	0.52275	6.77133	< 0.00001	3.91505	0.00018
Amyg_R_2_1	0.886 ± 0.008	0.873 ± 0.014	0.794 ± 0.012	23.17599	< 0.00001	0.81816	0.41543	6.45103	< 0.00001	3.60321	0.00053
Tha_L_8_1	0.881 ± 0.008	0.858 ± 0.019	0.791 ± 0.010	22.56622	< 0.00001	1.22476	0.22386	6.89477	< 0.00001	3.31397	0.00136
PhG_R_6_2	0.745 ± 0.008	0.717 ± 0.016	0.672 ± 0.008	20.71815	< 0.00001	1.77547	0.07920	6.6293	< 0.00001	2.74907	0.00733
PhG_R_6_3	1.060 ± 0.011	1.014 ± 0.025	0.954 ± 0.011	20.59200	< 0.00001	1.87852	0.06355	6.74265	< 0.00001	2.61339	0.01064
Hipp_L_2_1	0.993 ± 0.007	0.978 ± 0.014	0.911 ± 0.012	20.01990	< 0.00001	1.02713	0.30711	6.07115	< 0.00001	3.11746	0.00251
Tha_R_8_8	0.872 ± 0.007	0.850 ± 0.015	0.799 ± 0.009	19.79848	< 0.00001	1.48625	0.14071	6.28472	< 0.00001	2.88343	0.00501
Tha_L_8_8	0.213 ± 0.002	0.204 ± 0.004	0.193 ± 0.003	19.28024	< 0.00001	2.02410	0.04592	6.28866	< 0.00001	2.38611	0.0193
Amyg_R_2_2	0.876 ± 0.007	0.864 ± 0.012	0.804 ± 0.010	19.24375	< 0.00001	0.89500	0.37318	5.88859	< 0.00001	3.22866	0.00178
Tha_R_8_6	1.004 ± 0.009	0.989 ± 0.015	0.925 ± 0.009	18.81174	< 0.00001	0.78177	0.43640	5.92915	< 0.00001	3.59725	0.00055
Tha_L_8_6	0.894 ± 0.007	0.868 ± 0.015	0.825 ± 0.008	18.64184	< 0.00001	1.65133	0.10216	6.23128	< 0.00001	2.63323	0.01009
Tha_L_8_5	0.783 ± 0.006	0.758 ± 0.014	0.720 ± 0.009	17.60891	< 0.00001	1.87432	0.06413	6.04812	< 0.00001	2.29620	0.02419
Amyg_L_2_2	1.017 ± 0.008	1.009 ± 0.013	0.940 ± 0.012	17.01460	< 0.00001	0.53252	0.59568	5.46231	< 0.00001	3.17655	0.00209
Tha_R_8_5	0.844 ± 0.008	0.834 ± 0.013	0.780 ± 0.009	15.85335	< 0.00001	0.63783	0.52520	5.40814	< 0.00001	3.29199	0.00146
BG_R_6_5	0.612 ± 0.007	0.747 ± 0.030	0.666 ± 0.015	15.85256	< 0.00001	-6.41057	<0.00001	-3.33799	0.00110	2.60203	0.01097
MTG_R_4_4	0.826 ± 0.006	0.791 ± 0.010	0.776 ± 0.007	15.84700	< 0.00001	2.93371	0.00425	5.48661	< 0.00001	1.15667	0.25072
ITG_L_7_4	0.885 ± 0.008	0.883 ± 0.016	0.820 ± 0.009	15.80200	< 0.00001	0.12387	0.90170	5.38992	< 0.00001	3.68310	0.00041
FuG_R_3_2	0.577 ± 0.005	0.564 ± 0.011	0.533 ± 0.005	15.61783	< 0.00001	1.08429	0.28113	5.74139	< 0.00001	2.83167	0.00581

Amyg_L_2_1	0.955 ± 0.007	0.956 ± 0.014	0.881 ± 0.013	15.40595	< 0.00001	-0.02009	0.98402	5.07844	< 0.00001	3.20071	0.00194
FuG_R_3_3	0.747 ± 0.007	0.721 ± 0.013	0.693 ± 0.006	15.23303	< 0.00001	1.74519	0.08437	5.63068	< 0.00001	2.23271	0.02826
CG_R_7_2	0.650 ± 0.008	0.624 ± 0.015	0.585 ± 0.009	15.13797	< 0.00001	1.64007	0.10448	5.53393	< 0.00001	2.29765	0.02410
ITG_L_7_2	0.430 ± 0.006	0.404 ± 0.010	0.383 ± 0.006	15.08428	< 0.00001	2.03353	0.04494	5.5051	< 0.00001	1.93044	0.05697
INS_R_6_4	0.756 ± 0.008	0.768 ± 0.015	0.702 ± 0.007	15.00983	< 0.00001	-0.72862	0.46813	4.96958	< 0.00001	4.30433	0.00005
FuG_L_3_3	0.779 ± 0.007	0.773 ± 0.013	0.727 ± 0.007	15.00660	< 0.00001	0.43138	0.66722	5.34311	< 0.00001	3.37104	0.00114
FuG_L_3_1	0.752 ± 0.005	0.756 ± 0.010	0.705 ± 0.009	14.71222	< 0.00001	-0.32449	0.74632	4.93571	< 0.00001	3.29354	0.00146
STG_R_6_5	0.583 ± 0.006	0.587 ± 0.010	0.541 ± 0.006	14.67813	< 0.00001	-0.37497	0.70856	4.97965	< 0.00001	4.00343	0.00014
BG_R_6_3	0.736 ± 0.005	0.742 ± 0.010	0.696 ± 0.006	14.60654	< 0.00001	-0.58283	0.56147	4.92784	< 0.00001	3.68294	0.00041
IPL_L_6_1	0.495 ± 0.006	0.471 ± 0.009	0.454 ± 0.005	14.20192	< 0.00001	2.20578	0.02995	5.25747	< 0.00001	1.64543	0.10366
INS_L_6_4	0.748 ± 0.007	0.773 ± 0.015	0.703 ± 0.008	13.72173	< 0.00001	-1.68546	0.09536	4.19079	0.00005	4.33513	0.00004
PhG_L_6_3	1.093 ± 0.010	1.064 ± 0.019	1.012 ± 0.012	13.7038	< 0.00001	1.45373	0.14950	5.23272	< 0.00001	2.23311	0.02823
OrG_L_6_5	0.673 ± 0.006	0.654 ± 0.011	0.626 ± 0.007	13.67776	< 0.00001	1.64161	0.10416	5.27278	< 0.00001	2.07584	0.04100
MTG_R_4_2	0.641 ± 0.005	0.636 ± 0.012	0.600 ± 0.006	13.50060	< 0.00001	0.38261	0.70291	5.26724	< 0.00001	2.94246	0.00422
OrG_R_6_5	0.608 ± 0.006	0.579 ± 0.014	0.556 ± 0.007	13.46206	< 0.00001	2.14920	0.03430	5.33428	< 0.00001	1.54064	0.12721
STG_R_6_3	0.667 ± 0.005	0.667 ± 0.008	0.630 ± 0.006	12.97593	0.00001	0.06450	0.94872	4.68504	0.00001	3.37500	0.00112
MFG_R_7_4	0.568 ± 0.006	0.544 ± 0.007	0.526 ± 0.006	12.77163	0.00001	2.02393	0.04594	4.86516	< 0.00001	1.71959	0.08923
ITG_R_7_4	0.707 ± 0.007	0.701 ± 0.014	0.653 ± 0.009	12.60818	0.00001	0.45961	0.64690	4.86467	< 0.00001	2.89379	0.00486
IPL_L_6_5	0.758 ± 0.006	0.764 ± 0.009	0.721 ± 0.006	12.01352	0.00001	-0.52744	0.59918	4.33438	0.00003	3.69883	0.00039
INS_R_6_5	0.737 ± 0.008	0.712 ± 0.016	0.683 ± 0.007	11.97393	0.00001	1.57734	0.11823	5.05924	< 0.00001	1.88233	0.06330
OrG_L_6_1	0.562 ± 0.007	0.547 ± 0.012	0.519 ± 0.006	11.79271	0.00002	1.17755	0.24208	4.94782	< 0.00001	2.36161	0.02054
BG_L_6_3	0.822 ± 0.005	0.828 ± 0.011	0.787 ± 0.006	11.58230	0.00002	-0.60527	0.54653	4.43064	0.00002	3.48667	0.00078
STG_L_6_5	0.678 ± 0.006	0.685 ± 0.011	0.632 ± 0.009	11.56967	0.00002	-0.57313	0.56798	4.23658	0.00004	3.33417	0.00128
MTG_L_4_4	0.773 ± 0.007	0.754 ± 0.015	0.722 ± 0.007	11.46771	0.00002	1.21913	0.22598	4.91853	< 0.00001	2.16984	0.03288
Tha_R_8_2	0.152 ± 0.002	0.141 ± 0.004	0.138 ± 0.002	11.39499	0.00002	2.86693	0.00516	4.64962	0.00001	0.57235	0.56863
CG_R_7_7	0.868 ± 0.009	0.832 ± 0.015	0.805 ± 0.010	11.38250	0.00002	2.04808	0.04347	4.73270	0.00001	1.41044	0.16215
IPL_R_6_5	0.564 ± 0.005	0.557 ± 0.010	0.530 ± 0.005	11.3544	0.00003	0.75596	0.45165	4.77397	< 0.00001	2.60115	0.01100
INS_R_6_2	0.815 ± 0.008	0.810 ± 0.016	0.757 ± 0.009	11.30041	0.00003	0.24401	0.80778	4.58770	0.00001	2.92641	0.00442
PhG_L_6_1	0.640 ± 0.010	0.647 ± 0.021	0.574 ± 0.011	11.19943	0.00003	-0.30502	0.76105	4.42850	0.00002	3.34162	0.00125
CG_L_7_7	0.662 ± 0.007	0.634 ± 0.017	0.610 ± 0.007	11.16965	0.00003	1.72833	0.08736	4.98335	< 0.00001	1.55757	0.12314
BG_L_6_5	0.720 ± 0.009	0.850 ± 0.033	0.792 ± 0.020	10.88654	0.00004	-5.22451	<0.00001	-3.40723	0.00088	1.53613	0.12831

INS_L_6_3	0.889 ± 0.008	0.890 ± 0.016	0.838 ± 0.009	10.67451	0.00005	-0.00507	0.99596	4.42724	0.00002	3.03608	0.00320
INS_L_6_5	0.642 ± 0.007	0.626 ± 0.014	0.592 ± 0.008	10.59115	0.00005	1.09739	0.27540	4.65468	0.00001	2.11992	0.03700
IPL_L_6_4	0.562 ± 0.006	0.560 ± 0.011	0.525 ± 0.006	10.34441	0.00006	0.13233	0.89502	4.39882	0.00002	2.81255	0.00613
pSTS_R_2_1	0.770 ± 0.010	0.749 ± 0.014	0.713 ± 0.007	10.29610	0.00006	1.12948	0.26170	4.49270	0.00002	2.34344	0.02150
INS_L_6_6	0.816 ± 0.008	0.841 ± 0.016	0.773 ± 0.009	10.25803	0.00007	-1.44327	0.15242	3.59027	0.00047	3.89072	0.00020
PhG_R_6_1	0.643 ± 0.009	0.620 ± 0.023	0.572 ± 0.012	10.18467	0.00007	1.09278	0.27741	4.68521	0.00001	1.98231	0.05075
IFG_R_6_2	0.497 ± 0.007	0.467 ± 0.010	0.451 ± 0.008	10.13865	0.00007	2.21301	0.02943	4.30912	0.00003	1.10802	0.27105
ITG_R_7_3	0.691 ± 0.007	0.698 ± 0.013	0.652 ± 0.007	10.01257	0.00008	-0.47192	0.63812	4.07563	0.00008	3.37267	0.00113
IPL_L_6_3	0.546 ± 0.006	0.531 ± 0.010	0.509 ± 0.006	9.92649	0.00009	1.33724	0.18452	4.45701	0.00002	1.86726	0.06540
PhG_L_6_5	0.800 ± 0.007	0.807 ± 0.015	0.751 ± 0.010	9.79525	0.00010	-0.47149	0.63844	4.01335	0.00010	2.92265	0.00447
PhG_L_6_2	0.830 ± 0.007	0.817 ± 0.015	0.780 ± 0.009	9.77769	0.00010	0.90976	0.36538	4.40974	0.00002	2.12491	0.03657
IPL_R_6_4	0.562 ± 0.006	0.547 ± 0.011	0.526 ± 0.006	9.76858	0.00010	1.27144	0.20685	4.46738	0.00002	1.89821	0.06114
LOcC_L_4_4	0.382 ± 0.005	0.361 ± 0.008	0.355 ± 0.004	9.73465	0.00011	2.36949	0.01995	4.29965	0.00003	0.66747	0.50632
STG_L_6_1	0.688 ± 0.007	0.696 ± 0.012	0.643 ± 0.010	9.66633	0.00011	-0.56220	0.57538	3.86528	0.00017	2.99051	0.00366
IFG_R_6_4	0.462 ± 0.005	0.440 ± 0.009	0.429 ± 0.006	9.64062	0.00011	2.16486	0.03304	4.31297	0.00003	1.02572	0.30800
ITG_L_7_3	0.965 ± 0.007	0.984 ± 0.014	0.922 ± 0.010	9.56905	0.00012	-1.26284	0.20991	3.57521	0.00049	3.34632	0.00123
ITG_L_7_6	0.722 ± 0.007	0.730 ± 0.013	0.680 ± 0.008	9.38898	0.00014	-0.52233	0.60272	3.87040	0.00017	3.16460	0.00217
CG_R_7_1	0.708 ± 0.007	0.670 ± 0.013	0.663 ± 0.008	9.37380	0.00015	2.55123	0.01242	4.16960	0.00006	0.45393	0.65107
INS_L_6_1	0.596 ± 0.007	0.586 ± 0.013	0.552 ± 0.007	9.33652	0.00015	0.67450	0.50172	4.31016	0.00003	2.41072	0.01813
INS_R_6_3	0.929 ± 0.009	0.922 ± 0.017	0.875 ± 0.009	9.30913	0.00015	0.40790	0.68432	4.24501	0.00004	2.51890	0.01369
SFG_L_7_7	0.474 ± 0.004	0.465 ± 0.010	0.446 ± 0.004	9.29272	0.00016	0.94096	0.34924	4.54288	0.00001	2.05069	0.04345
OrG_L_6_3	0.703 ± 0.006	0.666 ± 0.011	0.666 ± 0.006	9.19082	0.00017	2.89304	0.00478	3.97718	0.00012	-0.01111	0.99116
pSTS_R_2_2	0.755 ± 0.009	0.738 ± 0.011	0.706 ± 0.007	9.15607	0.00018	0.97567	0.33184	4.13634	0.00006	2.31576	0.02304

Regional grey matter volumes of the three groups are shown as the mean ± SD (P <0.05, Bonferroni corrected). F, F value of the ANOVA; t, t score of the *post hoc* analysis; GMV, grey matter volume.

Amyg, amygdala; BG, basal ganglia; CG, cingulate gyrus; FuG, fusiform gyrus; Hipp, hippocampus; IFG, inferior frontal gyrus; INS, insular gyrus; IPL, inferior parietal lobule; ITG, inferior temporal gyrus; LOcC, lateral occipital cortex; MFG, middle frontal gyrus; MTG, middle temporal

gyrus; OrG, orbital gyrus; PhG, parahippocampal gyrus; PoG, postcentral gyrus; pSTS, posterior superior temporal sulcus; SFG, superior frontal gyrus; STG, superior temporal gyrus; Tha, thalamus; L, left; R, right. Detailed information on the brain regions is available at http://atlas.brainnetome.org.

Table S13. Regional cortical thickness that separated the two WMH groups in the logistic regression model.

Independent variables	β	SE	Wald	df	*P*	Odds ratio	95% CI
Age	0.127	0.057	5.061	1	0.024	1.136	(1.017, 1.269)
MFG_R_7_3	8.312	3.017	7.592	1	0.006	4070.957	(11.016, 1504451.742)
ITG_L_7_6	4.365	1.598	7.457	1	0.006	78.634	(3.428, 1803.63)
Constant	-39.539	10.989	12.947	1	< 0.001	$6.734*10^{-18}$	
Model χ^2	26.851, df = 3, *P* < 0.001						
Pseudo R^2	0.461						
Prediction accuracy	77.3%						

β, standardized regression coefficient; SE : standard error. MFG, middle frontal gyrus; ITG, inferior temporal gyrus; L, left; R, right. Detailed information on the brain regions is available at http://atlas.brainnetome.org.

The table shows the prediction performance of regional cortical thickness using logistic regression (forward) to classify the two WMH groups. Considering the relatively small samples with too many features, only regional cortical thicknesses that altered in the between-group comparisons were entered into the model. In addition, age, sex, and years of education were also entered into the models as variables. The results showed that a model with age and two regional cortical thickness variables separated the two WMH groups well (with a prediction accuracy of 77.3%).

Table S14. Regional grey matter volume that separated the two WMH groups in the logistic regression model.

Independent variables	β	SE	Wald	df	*P*	Odds ratio	95% CI
MFG_L_7_3	18.185	7.637	5.67	1	0.017	$7.902*10^{7}$	$(24.93, 2.504*10^{14})$
Tha_R_8_5	15.19	4.601	10.901	1	0.001	$3.954*10^{6}$	$(479.632, 3.260*10^{10})$
Constant	-13.561	3.999	11.5	1	0.001	$1.0*10^{-6}$	
Model χ^2	22.360, df = 2, $P < 0.001$						
Pseudo R^2	0.396						
Prediction accuracy	83.3%						

β, Standardized regression coefficient; SE, standard error. MFG, middle frontal gyrus; Tha, thalamus; L, left; R, right. Detailed information om the brain regions is available at http://atlas.brainnetome.org.

The table shows the predictive performance of regional grey matter volume using logistic regression (forward) to classify the two WMH groups. Considering the relatively small samples with too many features, only regional grey matter volume that altered in the between-group comparisons were entered into the model. In addition, age, sex, years of education, and TIV were also entered into the models as variables. The results showed that a model with two regional grey matter volume variables distinguished WMH-MCI patients from WMH-nCI patients with a high level of accuracy (83.3%).

Supplementary Figures

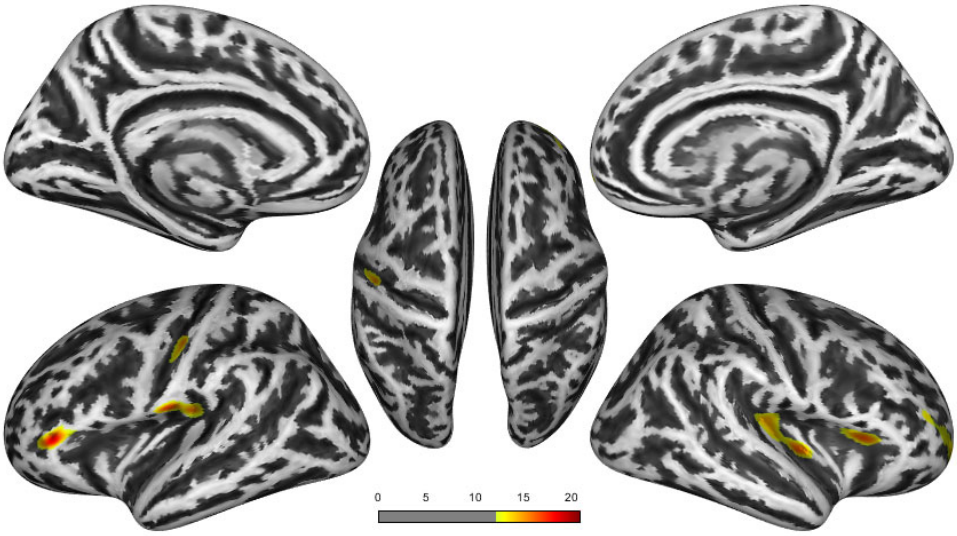

Fig. S1 ANOVA for cortical thickness across the three groups in the in-house dataset at the voxel level. All effects survived the false discovery rate (FDR) correction for multiple comparisons at the peak level (*P* <0.01). The underlying surface is the inflated average surface

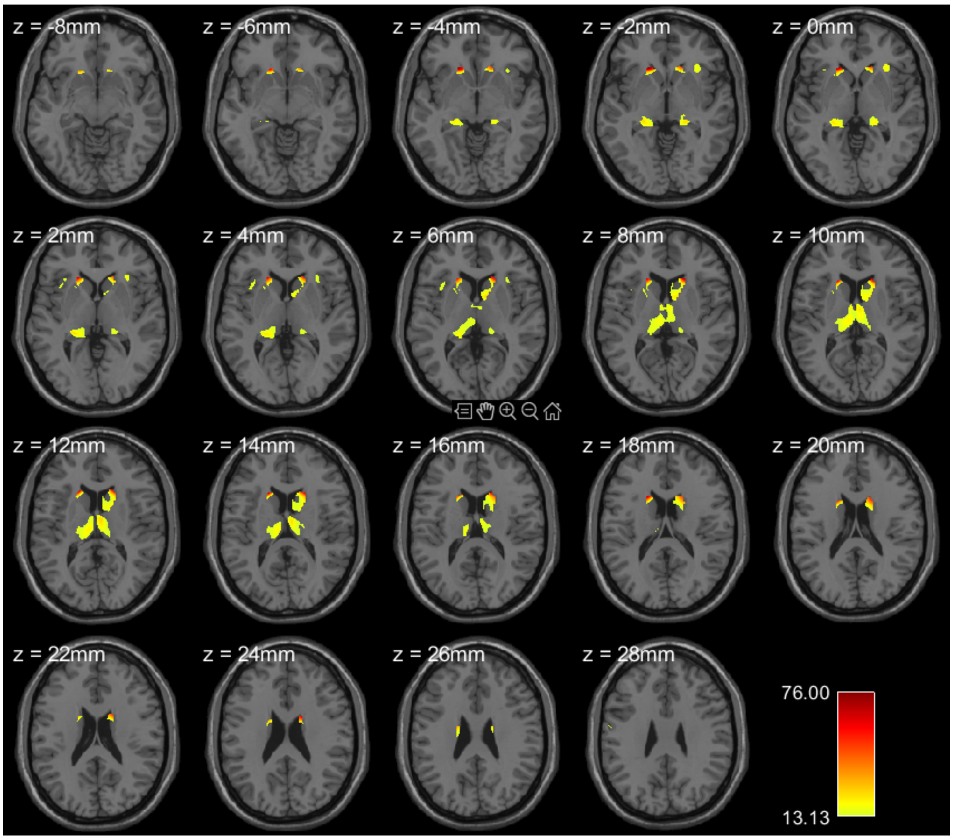

Fig. S2 ANOVA for grey matter volume across the three groups in the in-house dataset at the voxel level. All effects survived the false discovery rate (FDR) correction for multiple comparisons at the peak level (*P* <0.01)

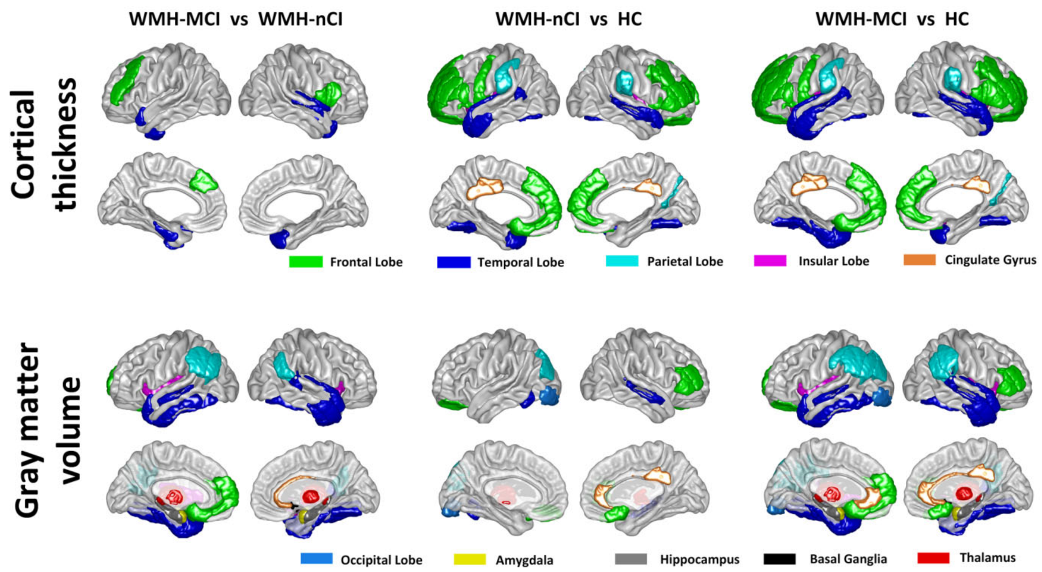

Fig. S3 Differences in grey matter volume and cortical thickness among the three groups in the replication dataset from ADNI based on the Human Brainnetome atlas (*P* <0.05, Bonferroni corrected)**.** The regions of altered grey matter volume or cortical thickness across the three groups are represented by different colors.

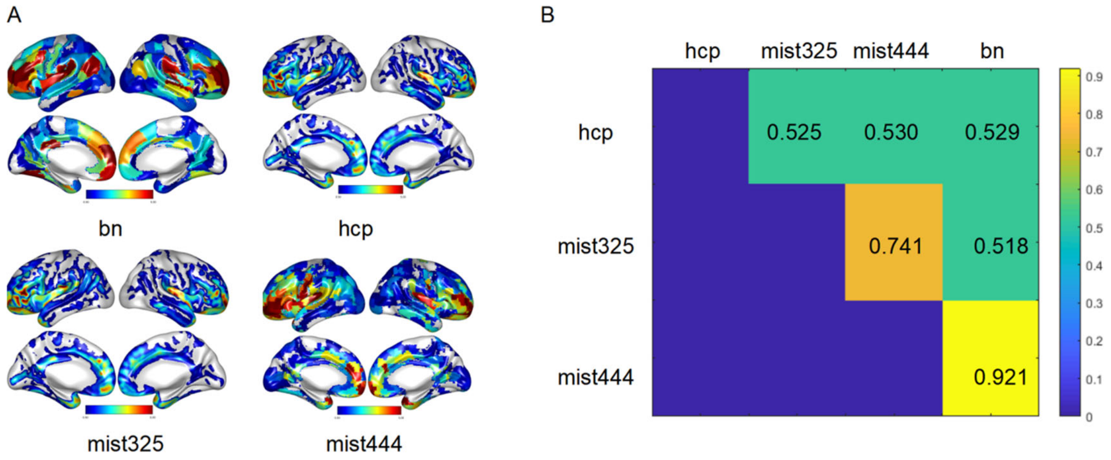

Fig. S4 Effect of different brain templates on main result of cortical thickness. A Alteration maps of cortical thickness among the three groups based on the Human Brainnetome (BN) Atlas of 272 regions, the Human Connectome Project (HCP) Atlas of 360 regions, and two atlases of the Multiresolution Intrinsic Segmentation Template (MIST) with 325 regions (MIST 325) and 444 regions (MIST 444). **B** Correlation map of –log p using the different templates. The colors represent different correlation coefficient values between –log p of the main results revealed by using each of the two templates. The alteration maps of cortical thickness among the three groups showed a very similar pattern using different atlases

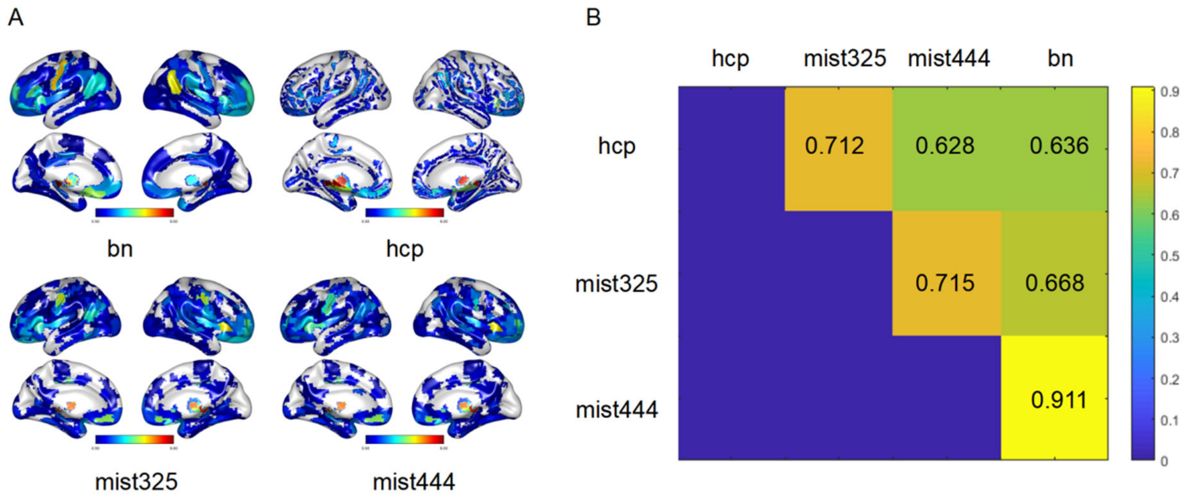

Fig. S5 Effect of different brain templates on the main result for grey matter volume. A Alteration maps of grey matter volume among the three groups based on the Human Brainnetome (BN) Atlas of 272 regions, the Human Connectome Project (HCP) Atlas of 360 regions, and two atlases of the Multiresolution Intrinsic Segmentation Template (MIST) with 325 regions (MIST 325) and 444 regions (MIST 444). **B** Correlation map between –log p in the main result using the different templates. The colors represent different correlation coefficient values between –log p of the main results revealed by using each of the two templates. The alteration maps of grey matter volume among the three groups showed a very similar pattern using different atlases.

References

[1] Papma JM, de Groot M, de Koning I, Mattace-Raso FU, van der Lugt A, Vernooij MW*, et al.* Cerebral small vessel disease affects white matter microstructure in mild cognitive impairment. Hum Brain Mapp 2014, 35: 2836–2851.

[2] Mirza SS, Ikram MA, Bos D, Mihaescu R, Hofman A, Tiemeier H. Mild cognitive impairment and risk of depression and anxiety: A population-based study. Alzheimers Dement 2017, 13: 130–139.

[3] Brissos S, Dias VV, Soeiro-de-Souza MG, Balanza-Martinez V, Kapczinski F. The impact of a history of psychotic symptoms on cognitive function in euthymic bipolar patients: a comparison with schizophrenic patients and healthy controls. Braz J Psychiatry 2011, 33: 353–361.

[4] Peterson KA, Savulich G, Jackson D, Killikelly C, Pickard JD, Sahakian BJ. The effect of shunt surgery on neuropsychological performance in normal pressure hydrocephalus: a systematic review and meta-analysis. J Neurol 2016, 263: 1669–1677.

[5] Jia J, Zhou A, Wei C, Jia X, Wang F, Li F*, et al.* The prevalence of mild cognitive impairment and its etiological subtypes in elderly Chinese. Alzheimers Dement 2014, 10: 439–447.

[6] Tuladhar AM, van Dijk E, Zwiers MP, van Norden AG, de Laat KF, Shumskaya E*, et al.* Structural network connectivity and cognition in cerebral small vessel disease. Hum Brain Mapp 2016, 37: 300–310.

[7] Petersen RC, Aisen PS, Beckett LA, Donohue MC, Gamst AC, Harvey DJ*, et al.*

Alzheimer's Disease Neuroimaging Initiative (ADNI): clinical characterization. Neurology 2010, 74: 201–209.
